# Hyperbranched Polyesters Based on Indole- and Lignin-Derived Monomeric Aromatic Aldehydes as Effective Nonionic Antimicrobial Coatings with Excellent Biocompatibility

**DOI:** 10.1021/acs.biomac.1c01186

**Published:** 2021-12-21

**Authors:** Xiaoya Li, Xiao Wang, Sathiyaraj Subramaniyan, Yang Liu, Jingyi Rao, Baozhong Zhang

**Affiliations:** † Centre for Analysis and Synthesis, Department of Chemistry, Lund University, P.O. Box 124, SE-22100 Lund, Sweden; ‡ Hubei Key Laboratory of Material Chemistry and Service Failure, Hubei Engineering Research Centre for Biomaterials and Medical Protective Materials, School of Chemistry and Chemical Engineering, Huazhong University of Science and Technology, Wuhan, Hubei 430074, People’s Republic of China; § Faculty of Medicine, Department of Clinical Sciences, Orthopedics, Lund University, 221 84 Lund, Sweden

## Abstract

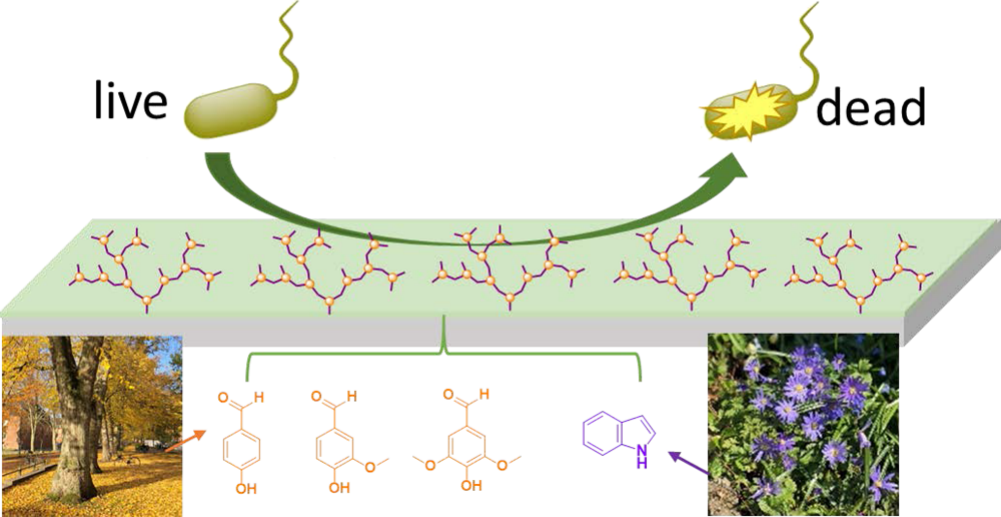

This research aims to investigate nonionic hyperbranched polyesters (HBPs) derived from indole and lignin resources as new nontoxic antimicrobial coatings. Three nonionic HBPs with zero to two methoxy ether substituents on each benzene ring in the polymer backbones were synthesized by melt-polycondensation of three corresponding AB_2_ monomers. The molecular structures and thermal properties of the obtained HBPs were characterized by gel permeation chromatography, nuclear magnetic resonance spectroscopy, Fourier transform infrared spectroscopy, thermogravimetric analysis, and differential scanning calorimetry analyses. These HBPs were conveniently spin-coated on a silicon substrate, which exhibited significant antibacterial effect against Gram-negative (*Escherichia coli* and *Pseudomonas aeruginosa*) and Gram-positive bacteria (*Staphylococcus aureus* and *Enterococcus faecalis*). The presence of methoxy substituents enhanced the antimicrobial effect, and the resulting polymers showed negligible leakage in water. Finally, the polymers with the methoxy functionality exhibited excellent biocompatibility according to the results of hemolysis and MTT assay, which may facilitate their biomedical applications.

## 1 Introduction

Antimicrobial polymers (AMPs) have received growing attention as potentially new coating materials for biomedical devices, due to their enhanced antimicrobial effects, lower toxicity, and nonleaching advantage compared to small molecular antimicrobials.^1−5^ Most reported AMPs contain positive charges, whose antimicrobial mechanism largely relies on their ionic interactions with negatively charged bacterial membranes.^6−9^ However, many ionic AMPs suffer from undesirable water solubility, eco-toxicity, poor compatibility with nonionic matrix materials, and fouling potential,^10−15^ which could limit their biomedical applications. Nonionic AMPs have potential to resolve these limitations, so they can form a new class of desirable coatings for various biomedical applications.^16,17^

Due to the lack of ionic interactions with bacterial membranes, nonionic AMPs usually contain certain functionalities (e.g., chlorine, phenol, and so forth) that can interact with bacterial membranes by, for example, hydrogen-bonding, hydrophobic, or dipole–dipole interactions.^17−21^ A smart strategy to design nonionic AMPs is to utilize naturally existing molecules with antimicrobial properties, such as curcumin, limonene, aspirin, indole, and so forth.^22−26^ Grafting such functionalities on linear polymer backbones can yield AMPs with an effective antimicrobial function.^27−32^ If such functionalities are densely grafted on highly branched polymers (e.g*.*, dendrimers, hyperbranched polymers, or HBPs), the interactions with bacterial membranes can be further enhanced, leading to more significant antibacterial effects.^7,8,16,18,31,33^ Such a dendritic enhancement of the antimicrobial effect has been frequently reported for ionic AMPs^34−36^ and less frequently reported for nonionic AMPs.^16,31,37^

When AMPs are used as coatings to protect the matrix material against bacteria,^38,39^ they could either prevent bacterial adhesion or kill the bacteria on contact. AMPs usually do not diffuse and release from the matrix and kill the surrounding bacteria (like small antibiotics or metal ions),^40,41^ which is due to their relatively large size and slow diffusion rate.^42−44^ AMP coatings with an anti-adhesion effect can be achieved by immobilizing antifouling agents such as polyethylene glycol and zwitterions.^45−48^ However, such coatings frequently suffer from harmful biofilm formation, due to the lack of bactericidal capabilities.^49,50^ Furthermore, the anti-adhesion effect could vary due to the changes in surface morphology and perfection.^51^ As such, it is advantageous for AMP coatings to exert a contact-killing effect. Contact killing is commonly achieved by using cationic agents (e.g., quaternary ammoniums, chitosan, peptides, cationic polymers, and so forth).^5,14,52−59^ However, nonionic AMPs with contact-killing capabilities were rarely investigated toward coating applications. To our knowledge, only a few nonionic polyphenolics have been reported so far.^20,21^ The design principles and structure–property relationships of nonionic AMP coatings remained largely unknown.

Herein, we present the synthesis of three bio-based nonionic hyperbranched polyesters using three AB_2_ monomers derived from various indole- and lignin-based monomeric molecules (methyl indole-5-carboxylate, 4-hydroxybenzaldehyde, vanillin, and syringaldehyde). The molecular and thermal properties, as well as the cytotoxicity of the obtained HBPs, were characterized. Their contact-killing antibacterial effect against two Gram-negative (*Escherichia coli* and *Pseudomonas aeruginosa*) and two Gram-positive bacteria (*Staphylococcus aureus* and *Enterococcus faecalis*) when used as coatings was also demonstrated.

## 2 Experimental Section

### 2.1 Chemicals and Materials

4-Hydroxybenzaldehyde, vanillin, syringaldehyde, ethylene carbonate, potassium carbonate (K_2_CO_3_), methyl indole-5-carboxylate, iodine (I_2_), and dibutyltin(IV) oxide (DBTO) were purchased from Sigma-Aldrich. Tetrahydrofuran (THF), *N*,*N*-dimethylformamide (DMF), *N*,*N*-dimethylacetamide (DMAc), 1,4-dioxane, chloroform, dichloromethane, dimethyl sulfoxide (DMSO), ethanol, methanol, acetone, acetonitrile, ethyl acetate (EtOAc), *n*-heptane, xylene, and Na_2_SO_4_ were purchased from VWR Chemicals. Tryptic soy broth (TSB), phosphate-buffered saline (PBS), tryptic soy agar (TSA), sterile sheep’s blood, *Staphylococcus aureus* ATCC 6538 (*S. aureus*), *Enterococcus faecalis* ATCC 29212 (*E. faecalis*), *Escherichia coli* ATCC 25922 (*E. coli*), and *Pseudomonas aeruginosa* ATCC 27853 (*P. aeruginosa*) were purchased from commercial sources. All chemicals were used as received without purification.

### 2.2 Synthesis

#### 2.2.1 General Procedure for Synthesis of **3a–c**

A solution of **1a–c** (4-hydroxybenzaldehyde, vanillin, and syringaldehyde, respectively, 10.0 mmol, 1.00 equiv) and K_2_CO_3_ (15.0 mmol, 1.50 equiv) in 50 mL of DMF was added into a 100 mL round-bottomed flask and stirred with N_2_ flow. Then, ethylene carbonate (11.0 mmol, 1.10 equiv) was added dropwise, and the reaction mixture was heated at 100 °C with refluxing. After 12 h, the reaction mixture was cooled to room temperature and poured into EtOAc (100 mL) before water (100 mL) was added. The aqueous phase was separated and then extracted with EtOAc (2× 50 mL). The organic phases were combined and washed with water (3× 50 mL), brine (50 mL), dried over Na_2_SO_4_, and concentrated under reduced pressure to yield **3a–c**.

##### 2.2.1.1 4-(2-Hydroxy-ethoxy)-benzaldehyde (**3a**)

White solid (50% yield), ^1^H NMR (400.13 MHz, DMSO-*d*_6_): δ ppm 9.87 (s, 1H, CHO), 7.87 (d, 2H, Ar), 7.13 (d, 2H, Ar), 4.96 (t, 1H, OH), 4.11 (t, 2H, OC*H*_*2*_CH_2_OH), 3.75 (m, 2H, OCH_2_C*H*_*2*_OH). ^13^C NMR (100.61 MHz, DMSO-*d*_6_): δ ppm 191.76, 164.21, 132.28, 130.12, 115.42, 70.53, 59.84. HRMS (ESI^+^, *m*/*z*): exact mass calcd for C_9_H_11_O_3_^+^, 167.0708; found, 167.0706.

##### 2.2.1.2 4-(2-Hydroxyethoxy)-2-methoxybenzaldehyde (**3b**)

Light yellow solid (45% yield), ^1^H NMR (400.13 MHz, DMSO-*d*_6_): δ ppm 9.85 (s, 1H, CHO), 7.54 (d, 1H, Ar), 7.41 (s, 1H, Ar), 7.20 (d, 1H, Ar), 4.94 (t, 1H, OH), 4.11 (t, 2H, OC*H*_*2*_CH_2_OH), 3.85 (s, 3H, OCH_3_) 3.77 (m, 2H, OCH_2_C*H*_*2*_OH). ^13^C NMR (100.61 MHz, DMSO-*d*_6_): δ ppm 191.83, 154.15, 149.70, 130.04, 126.53, 112.56, 110.07, 70.88, 59.80, 55.92. HRMS (ESI^+^, *m*/*z*): exact mass calcd for C_10_H_13_O_4_^+^, 197.0814; found, 196.0812.

##### 2.2.1.3 4-(2-Hydroxyethoxy)-2,6-dimethoxybenzaldehyde (**3c**)

Light yellow solid (42% yield), ^1^H NMR (400.13 MHz, DMSO-*d*_6_): δ ppm 9.89 (s, 1H, CHO), 7.27 (s, 2H, Ar), 4.64 (t, 1H, OH), 3.99 (t, 2H, OC*H*_*2*_CH_2_OH), 3.87 (s, 6H, OCH_3_) 3.65 (m, 2H, OCH_2_C*H*_*2*_OH). ^13^C NMR (100.61 MHz, DMSO-*d*_6_): δ ppm 192.32, 153.81, 142.62, 131.99, 107.27, 74.77, 60.74, 50.61. HRMS (ESI^+^, *m*/*z*): exact mass calcd for C_11_H_15_O_5_^+^, 227.0919; found, 227.0918.

#### 2.2.2 Synthesis of Monomers **5a–c**

To a well-stirred solution of **3a–c** (0.200 mol, 1.00 equiv) and indole-5-carboxylate (0.400 mol, 2.00 equiv) in acetonitrile (50 mL) was added I_2_ (catalytic amount) in a 100 mL round-bottomed flask with N_2_ flow. The reaction mixture was stirred at room temperature for 8 h. Afterward, the reaction mixture was poured into EtOAc (100 mL), followed by the addition of water (50 mL). The aqueous phase was separated and extracted with EtOAc (2× 50 mL). The combined organic phase was washed with water (3× 50 mL), brine (50 mL), dried over Na_2_SO_4_, and concentrated under reduced pressure to yield the corresponding monomers **5a–c**.

**5a**: brown solid (90% yield), ^1^H NMR (400.13 MHz, DMSO-*d*_6_): δ ppm 11.26 (s, 2H, NH), 8.02 (s, 2H, Ar), 7.70 (m, 2H, Ar), 7.46 (d, 2H, Ar), 7.23 (d, 2H, Ar), 6.85 (m, 4H, Ar), 5.95 (s, 1H, CH), 4.87 (t, 1H, OH), 3.94 (t, 2H, OC*H*_*2*_CH_2_OH) 3.76 (s, 6H, COOC*H*_*3*_), 3.70 (m, 2H, OCH_2_C*H*_*2*_OH); ^13^C NMR (100.61 MHz, DMSO-*d*_6_): δ ppm 167.67, 157.43, 139.77, 136.55, 129.61, 126.57, 125.94, 122.52, 122.18, 120.30, 114.58, 111.98, 69.81, 60.07, 52.05, 38.61. HRMS (ESI^+^, *m*/*z*): exact mass calcd for C_29_H_27_N_2_O_6_^+^, 499.1869; found, 499.1862.

**5b**: brown solid (89% yield), ^1^H NMR (400.13 MHz, DMSO-*d*_6_): δ ppm 11.25 (s, 2H, NH), 8.05 (s, 2H, Ar), 7.71 (d, 2H, Ar), 7.45 (d, 2H, Ar), 7.03 (s, 1H, Ar), 6.94–6.75 (m, 4H, Ar), 5.94 (s, 1H, CH), 4.82 (t, 1H, OH), 3.92 (t, 2H, OC*H*_*2*_CH_2_OH) 3.77 (s, 3H, COOC*H*_*3*_), 3.69 (m, 2H, OCH_2_C*H*_*2*_OH), 3.60 (s, 3H, OC*H*_*3*_); ^13^C NMR (100.61 MHz, DMSO-*d*_6_): δ ppm 167.67, 149.09, 146.94, 139.76, 137.25, 126.60, 125.92, 122.50, 122.22, 120.61, 120.23, 120.18, 113.32, 113.14, 111.98, 70.54, 60.08, 55.91, 52.05, 38.86. HRMS (ESI^+^, *m*/*z*): exact mass calcd for C_30_H_29_N_2_O_7_^+^, 529.1975; found, 529.1983.

**5c**: brown solid (91% yield), ^1^H NMR (400.13 MHz, DMSO-*d*_6_): δ ppm 11.28 (s, 2H, NH), 8.10 (s, 2H, Ar), 7.71 (d, 2H, Ar), 7.45 (d, 2H, Ar), 7.01 (s, 2H, Ar), 6.75 (s, 2H, Ar), 5.96 (s, 1H, CH), 4.53 (t, 1H, OH), 3.84 (t, 2H, OC*H*_*2*_CH_2_OH) 3.78 (s, 6H, COOC*H*_*3*_), 3.68 (s, 6H, OC*H*_*3*_), 3.60 (m, 2H, OCH_2_C*H*_*2*_OH); ^13^C NMR (100.61 MHz, DMSO-*d*_6_): δ ppm 167.68, 153.15, 140.30, 139.72, 135.47, 126.59, 125.94, 122.51, 122.25, 120.27, 119.82, 112.01, 106.48, 74.52, 60.67, 56.43, 52.05, 39.96. HRMS (ESI^+^, *m*/*z*): exact mass calcd for C_31_H_31_N_2_O_8_^+^, 559.2080; found, 559.2072.

#### 2.2.3 Polymerization of HBPs (**P5a–c**)

Monomers **5a–c** (500 mg), DBTO (25 mg), and xylene (10 mL) were added to a two-necked 50 mL round-bottomed flask equipped with a mechanical stirrer. After being stirred for 30 min under a N_2_ flow, the temperature was increased up to 165 °C and stirred again for 8 h. Afterward, the reaction mixture was cooled to room temperature and xylene was removed under reduced pressure. The obtained solid was dissolved in THF (5 mL) and precipitated in *n*-heptane (100 mL). The precipitates were collected by gravity filtration, redissolved in THF (3 mL), and reprecipitated in cold chloroform (100 mL) to yield **P5a–c**.

**P5a**: brown solid (36% yield), ^1^H NMR (400.13 MHz, DMSO-*d*_6_): δ ppm 11.24 (br, 2H, NH), 8.01 (br, 2H, Ar), 7.68 (br, 2H, Ar), 7.40 (br, 2H, Ar), 7.21 (br, 2H, Ar), 6.83 (br, 4H, Ar), 5.93 (br, 1H, CH), 4.41 (br, 2H, OC*H*_*2*_CH_2_OH), 4.13 (br, 2H, OCH_2_C*H*_*2*_OH), 3.71 (br, COOC*H*_*3*_). ^13^C NMR (100.61 MHz, DMSO-*d*_6_): δ ppm 167.67, 167.16, 156.97, 139.85, 139.77, 136.92, 129.66, 126.61, 126.56, 125.95, 122.55, 122.16, 120.35, 120.27, 120.13, 114.66, 111.96, 67.48, 66.22, 52.00, 38.62.

**P5b**: brown solid (35% yield), ^1^H NMR (400.13 MHz, DMSO-*d*_6_): δ ppm 11.26 (br, 2H, NH), 8.06 (br, 2H, Ar), 7.70 (br, 2H, Ar), 7.43 (br, 2H, Ar), 7.03 (br, 1H, Ar), 6.95–6.72 (br, 4H, Ar), 5.95 (br, 1H, CH), 4.44 (br, 2H, OC*H*_*2*_CH_2_OH), 4.17 (br, 2H, OCH_2_C*H*_*2*_OH), 3.74 (br, COOC*H*_*3*_), 3.59 (br, OC*H*_*3*_). ^13^C NMR (100.61 MHz, DMSO-*d*_6_): δ ppm 167.66, 167.21, 149.31, 146.53, 139.82, 139.75, 126.58, 125.94, 122.53, 122.19, 111.98, 67.32, 63.34, 55.96, 52.00, 39.11.

**P5c**: brown solid (40% yield), ^1^H NMR (400.13 MHz, DMSO-*d*_6_): δ ppm 11.23 (br, 2H, NH), 8.09 (br, 2H, Ar), 7.69 (br, 2H, Ar), 7.41 (br, 2H, Ar), 6.96 (br, 1H, Ar), 6.71 (br, 1H, Ar), 5.94 (br, 1H, CH), 4.34 (br, 2H, OC*H*_*2*_CH_2_OH), 4.08 (br, 2H, OCH_2_C*H*_*2*_OH), 3.69 (br, COOC*H*_*3*_), 3.52 (br, OC*H*_*3*_). ^13^C NMR (100.61 MHz, DMSO-*d*_6_): δ ppm 167.66, 167.22, 153.16, 140.57, 139.72, 135.04, 126.58, 125.94, 122.52, 122.33, 122.23, 120.49, 120.27, 119.81, 112.00, 111.82, 106.32, 70.95, 63.89, 56.24, 51.97, 40.89.

### 2.3 Measurements

Nuclear magnetic resonance (NMR) spectra were recorded on a Bruker DRX400 spectrometer at a proton frequency of 400.13 MHz and a carbon frequency of 100.61 MHz. Fourier transform infrared (FTIR) spectra were obtained with an attenuated total reflection setup using a Bruker Alpha FTIR spectrometer. Differential scanning calorimetry (DSC) measurements were performed using a TA Instruments DSC Q2000. The samples were studied with a heating rate of 10 °C min^–1^ under nitrogen with a purge rate of 50 mL min^–1^. The *T*_g_ was taken as the midpoint of the endothermic step-change observed during the second heating run. Thermogravimetric analysis (TGA) was performed under a nitrogen atmosphere with a thermogravimetric analyzer (TA Instrument Q500) at a heating rate 10 °C/min. Gel permeation chromatography (GPC) was carried out with 2xPL-Gel Mix-B LS column and OmniSEC triple detectors (refractive index, viscosity, and light scattering). All measurements were carried out at 35 °C at a concentration of 3 mg mL^–1^ using THF as the eluent, and at an elution rate of 1 mL min^–1^. Calibration was performed with a polystyrene standard sample (*M*_n_ = 96 kg mol^–1^ from Polymer Laboratories). High-resolution mass spectrometry (HRMS) was performed by direct infusion on a Water Xevo-G2 QTOF mass spectrometer using electrospray ionization. The optical density (OD) values were characterized by a microplate reader (MultiSkan, ND2k). SEM measurements were performed by a field-emission-scanning electron microscope (Hitachi SU8010). UV spectra were recorded by an ultraviolet–visible spectrophotometer (HTH HB-7). The thickness of coating was determined by the ellipsometry (SE-VM, Wuhan Eoptics Technology Co., Ltd.).

### 2.4 Preparation of Monomer or HBP Coatings

Silicon wafers (1 cm × 1 cm) were pre-treated with a piranha solution (98% sulfuric acid and 30% hydrogen peroxide, 7:3 v/v) for 30 min, then rinsed thoroughly with deionized water, and dried with nitrogen flow. Monomer (**5a–c**) or HBP (**P5a–c**) coatings were prepared by spin-coating (6000 rpm) from 20 μL of DMSO solutions (40 mg/mL) onto the silicon substrates. All coating samples were dried in a vacuum oven overnight at room temperature.

### 2.5 Antimicrobial Tests

The bactericidal potency of coatings was determined by following a contact protocol.^60−62^ Bacterial cells were grown overnight at 37 °C in a TSB medium to a mid-log phase and re-suspended in PBS to 1 × 10^6^ colony forming units per mL (CFU/mL). 10 μL of inoculum suspension was first spread on the uncoated (control), monomer-, or HBP-coated silicon wafer, then immediately covered with another piece of control or coated wafer. After incubation at 37 °C for 24 h, the wafer samples were transferred into 400 μL of the PBS solution bath and washed vigorously for 10 min. The surviving bacteria were plated on a TSA Petri dish with 100-fold serial dilutions and incubated at 37 °C for another 24 h. By counting the number of colonies on each plate, the survival numbers of bacteria were presented as log (cfu/mL). Each experiment was performed at least thrice.

Apart from this, the antibacterial performance of the coated silicon wafers was retested (second cycle). Specifically, after the antibacterial study against *E. coli*, the coated wafers were directly washed with PBS and water and then dried in a vacuum oven overnight. The antibacterial test against *E. coli* was again carried out as described above. Each experiment was performed thrice.

The leaching behavior of antibacterial agents from the coatings was examined by the zone of inhibition test and UV–vis spectrophotometry. Filter disks (6 mm in diameter) were immersed in the solutions of monomers or HBPs in DMSO (1 mg/mL) and then placed onto the sterilized TSA plates which were inoculated with bacterial cells (100 μL, 1 × 10^7^ CFU/mL) in advance. The solution of gentamycin in DMSO (1 mg/mL) and pure DMSO were used as controls. After 24 h of incubation at 37 °C, the possible zone of inhibition was recorded. The process was repeated three times to ensure the accuracy. For the UV–vis measurement, the coated wafer was immersed into a 1 mL H_2_O bath under shaking at 37 °C for 5 days. The UV–vis spectra of the aqueous phase were then measured. The control solutions were prepared by first dissolving the monomers or HBP polymers in DMSO then diluting in water to fix the final concentration at 0.1 mg/mL (DMSO/H_2_O = 1:9 v/v). Each sample was measured three times.

### 2.6 SEM Imaging

To observe the morphology of bacteria on HBP coatings, **P5c**-coated wafer was used as the representative sample and the uncoated wafer was used as the control. The antibacterial test against *E. coli* was carried out as described before. Afterward, the bacteria cells on **P5c**-coating were fixed in the glutaraldehyde solution (pH 7.2, 2.5%) for 2 h at room temperature. The bacterial cells were then dehydrated using gradient ethanol solutions (20, 50, 70, 80, 90, and 100% v/v in water) and dried in a vacuum oven. All samples were coated with gold using a Denton Dest II Sputter-Coater for 15 s and observed by FE-SEM.

To evaluate the antibiofouling effect, 10 μL of the *E. coli* suspension (1 × 10^6^ CFU/mL) was first spread on the uncoated (control) or HBP-coated wafers as described above. After incubation at 37 °C for 24 h, the wafers were simply washed by PBS and water and then dried in a vacuum oven overnight at room temperature. The surface of the coated wafers was examined using FE-SEM operated at 3 kV.

### 2.7 Thickness Analysis

The thickness of coatings was determined by the ellipsometry, including the freshly prepared coatings and the coated wafers after antibacterial tests. Four random positions on each wafer were measured, and the results were averaged. Each sample was measured three times.

### 2.8 Hemolysis Tests

Hemolytic activity was characterized with sheep’s blood. Red blood cells (RBCs) were pelletized by centrifuging 1 mL of the blood and washing the pellet four times with PBS (pH = 7.4). A 10 μL of the RBC suspension was first spread on the uncoated (control), monomer-, or HBP-coated silicon wafer, then immediately covered with another piece of control or coated wafer. After incubation at room temperature for 2 h, the wafer samples were transferred into a 490 μL of PBS or deionized water solution bath and washed vigorously for 10 min. For the uncoated wafers, the positive control was washed with deionized water and the negative control was washed with PBS. 100 μL of the diluted solution was transferred to a new 96 well plate and the OD at 540 nm was measured. The hemolysis percentage was calculated by following equation.
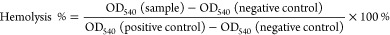


### 2.9 MTT Assay

The MG-63 osteoblast-like human cells were cultured in Dulbecco’s modified Eagle media supplemented with 10% fetal bovine serum, 1% penicillin, and 1% streptomycin in a humidified incubator at 37 °C. The medium was replaced every 2 days. Cells were trypsinized and centrifuged at 400*g* for 4 min to get a concentrated cell pellet when the confluence reached 80%. 1 × 10^4^ cells/well were seeded on a 96-well plate and cultured for 24 h before adding the materials. Test compounds (negative control, **5a–c**, and **P5a–c**) dissolved in DMSO were then added to the cell culture at a final DMSO concentration of 1% (v/v). Fresh culture medium without the tested samples was used as a negative control, and each sample was replicated in four wells. After being cultured for 24 h, the cell culture medium was discarded and the cells were washed with phosphate buffer. The MTT working solution (0.5 mg/mL) was added to the cells and incubated for 2 h at 37 °C, after which DMSO (200 μL/well) was added to the reaction products for 10 min. The solubilized contents were pipetted and transferred into a clear bottom 96-well plate. Absorbance was determined by spectrophotometry at 600 nm wavelength.

## 3 Results and Discussion

### 3.1 Synthesis of Monomers and HBPs

**Figure 1:**
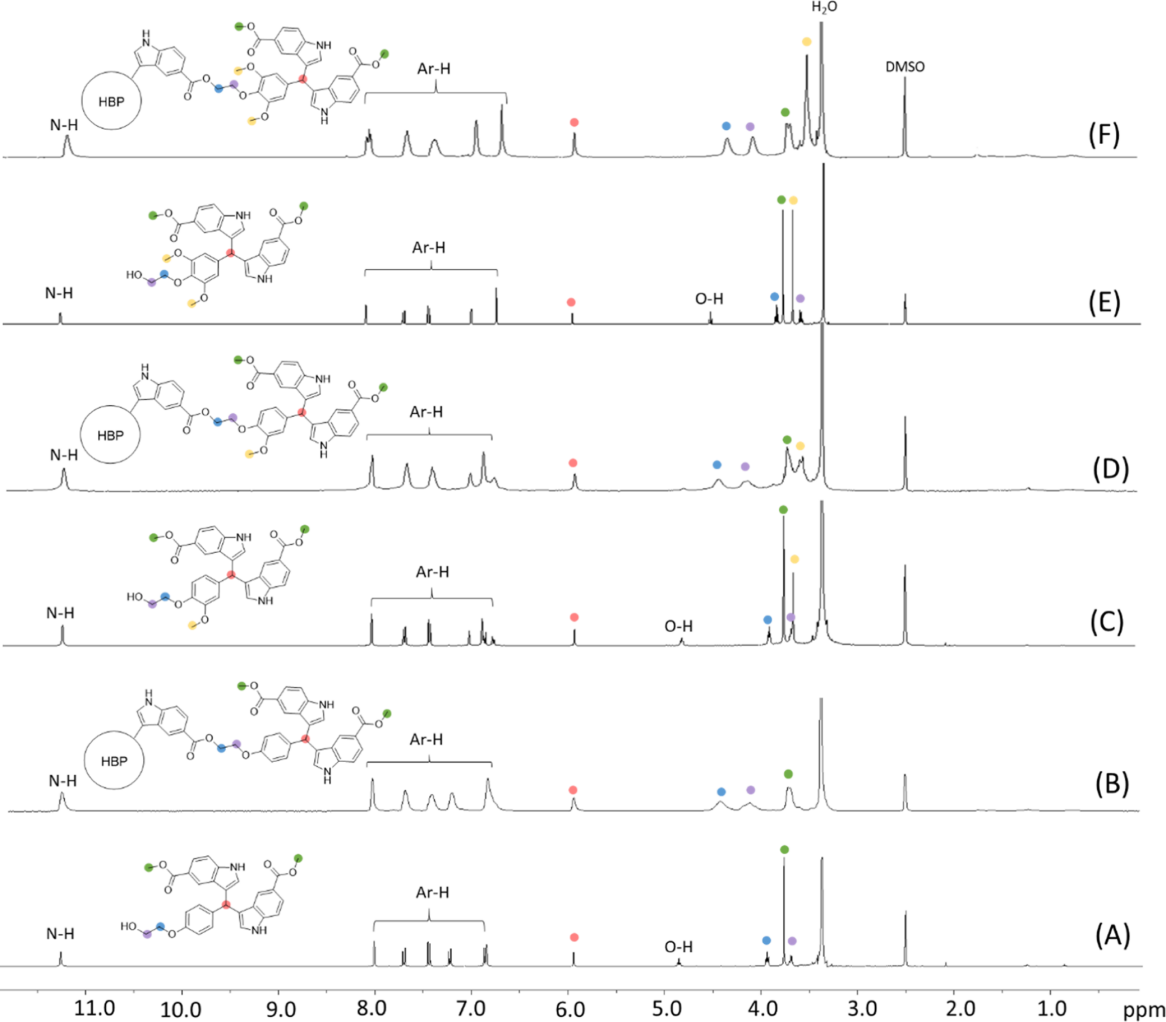
^1^H NMR spectra of (A) **5a**, (B) **P5a**, (C) **5b**, (D) **P5b**, (E) **5c**, and (F) **P5c** in DMSO-*d*_6_.

**Scheme 1:**
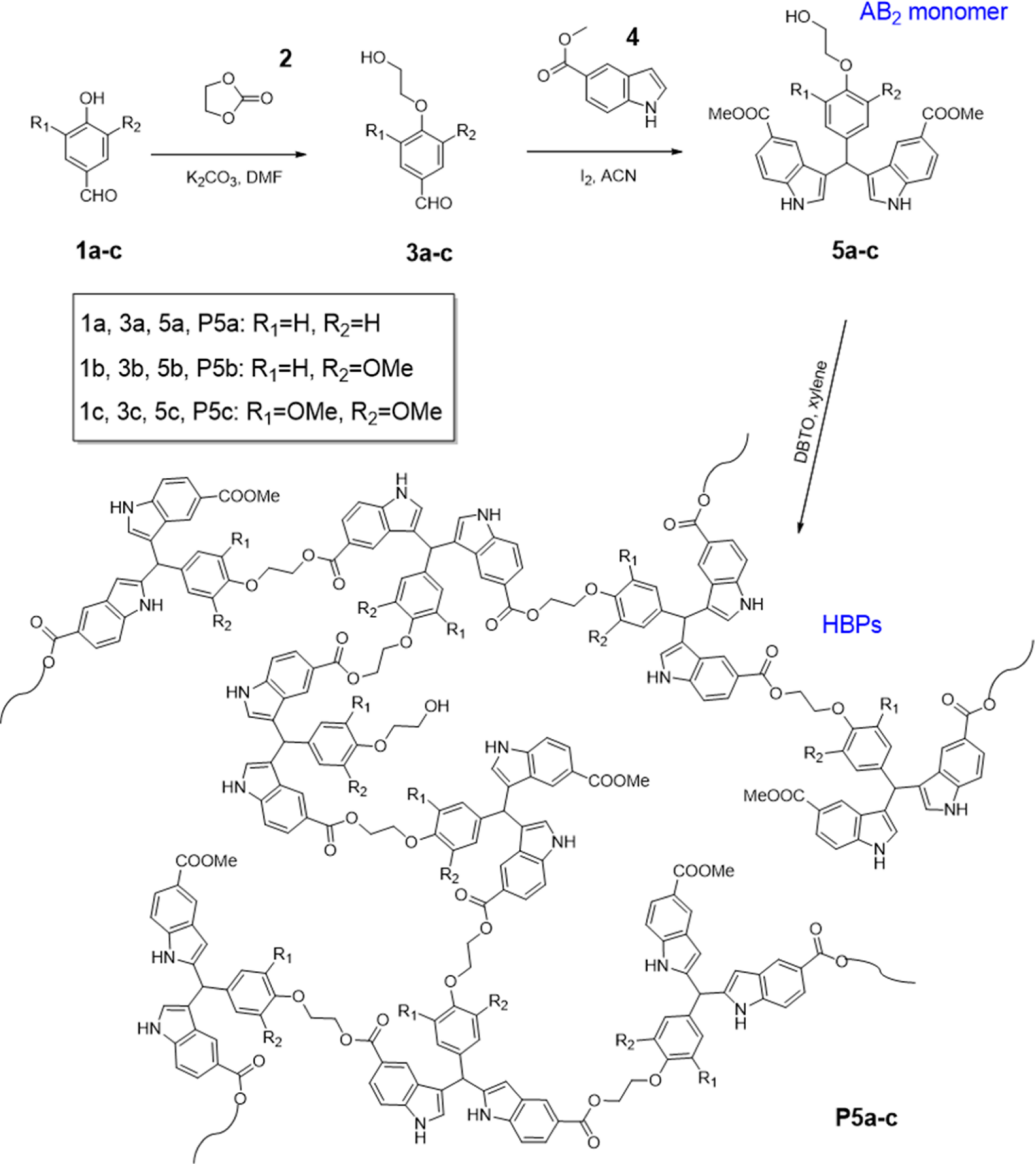
Synthesis of AB_2_ Monomers **5a–c** and HBPs (**P5a–c**)

The three AB_2_-type monomers (one OH and two COOMe groups) with a bis-indole structure (**5a–c**, Scheme 1) were synthesized in two steps from lignin-derived aromatic aldehydes (**1a–c**, Scheme 1). First, the phenolic groups of **1a–c** were reacted under mild basic conditions with ethylene carbonate (**2**), a green reagent, yielding the corresponding primary alcohols **3a–c**. Afterward, the aldehyde groups of **3a–c** were reacted with indole carboxylate **4** at the three position on indole rings according to an iodine-catalyzed protocol,^63^ yielding the corresponding AB_2_ monomers **5a–c** in ∼90% yields and good purity (according to ^1^H NMR spectra, Figure 1A,C,E).

The obtained AB_2_ monomers **5a–c** were polymerized by bulk condensation using a DBTO catalyst at 165 °C,^64,65^ yielding HBPs **P5a–c**, respectively. A small amount of xylene was added in the polymerization mixture to facilitate heat transfer and removal of the condensed methanol.^66^ An increased reaction temperature to 180 °C resulted in partial insolubility in THF due to cross-linking. Even a higher temperature (200 °C) led to coloration and char formation during the polymerization. After the polymerization, two straightforward precipitations of the crude polymer solution dissolved in THF into *n*-heptane and then into chloroform were carried out to yield pure polymers **P5a–c**. The obtained HBPs generally showed good solubility in polar aprotic solvents (e.g., DMSO, DMF, DMAc, and THF, Table S1, Supporting Information), which could facilitate their characterization and processing by spin-coating from their solutions.

### 3.2 Molecular Characterization

The molar masses of **P5a–c** were desirable in the medium–low range (∼3000–4500 g mol^–1^) according to the GPC results (Table 1). This range of molecular weight is desirable for the intended antimicrobial applications because a too high molecular weight could lead to decreased antimicrobial activity.^36^ It was also observed that upon an increased number of methoxy groups in the polymers (i.e., from **P5a** to **P5c**), the molecular weight showed a slightly decreasing trend. This may suggest that the presence of methoxy groups in monomers could lower their reactivity under the polymerization conditions, likely due to steric hindrance. In the meantime, the yields of these polymerizations were generally low (35–40%), which indicated the occurrence of fractionation due to different solubility of the crude products during purification. Such fractionation may lead to a change of the observed molecular weight after purification. Furthermore, the obtained monomers and polymers were characterized by ^1^H NMR spectroscopy (Figure 1). All the proton signals for the monomers (**5a–c**) were unambiguously assigned (Figure 1A,C,E), including the NH signals (11.26, 11.25, and 11.28 ppm for **5a–c**, respectively), the aromatic signals (∼8.10–6.75 ppm), the signals for the central CH (5.95, 5.94, and 5.96 ppm for **5a–c**, respectively), the OH signals (4.87, 4.82, and 4.53 ppm for **5a–c**, respectively), the two ethylene “bridge” signals (3.94, 3.92, and 3.84 ppm next to aromatic ether unit and 3.70, 3.69, and 3.60 ppm next to the OH group for **5a–c**, respectively), methyl ester signals (3.76, 3.77, and 3.78 ppm for **5a–c**, respectively), and the methoxy signals (3.60 ppm for **5b–c**). After polymerization, the ^1^H NMR spectra of the resulting polymers displayed broadened signals (Figure 1B,D,F), which indicated the formation of polymers. The OH signals in the ^1^H NMR spectra of monomers were not observed, which confirmed monomer consumption (note, there is only one OH group present in **P5a–c**, which may be too small to be observed). Furthermore, the ethylene “bridge” signals showed significant downfield shifts in the polymers compared to that of the corresponding monomers, which was consistent with the formation of electron-withdrawing ester bonds. All the other signals (i.e., the NH signal, aromatic signals, CH, and OCH_3_ signals) remained after the polymerizations without a significant change in the chemical shifts because they were located relatively far away from the reaction sites (esterification).

**Table 1:** Molecular and Thermal Properties of HBPs (**P5a–c**)^a^

	*M*_n_ (g mol^–1^)	*M*_w_ (g mol^–1^)	PDI	*T*_g_ (°C)	*T*_10_ (°C)	*T*_max_ (°C)	CY (%)
**P5a**	4 494	14 435	3.2	223	354	290, 402	60
**P5b**	3 761	12 920	3.5	213	317	300, 406	50
**P5c**	3 282	11 443	3.5	209	318	325, 422	50

a *M*_n_, *M*_w_, and PDI were determined by GPC in THF. *T*_g_ (glass-transition temperature) was measured from the second heating DSC curve and *T*_10_ and *T*_max_ are the temperatures for 10% weight loss and maximum decomposition rates, respectively, according to the TGA data. Char yield (CY) at 600 °C was measured by TGA.

**Figure 2:**
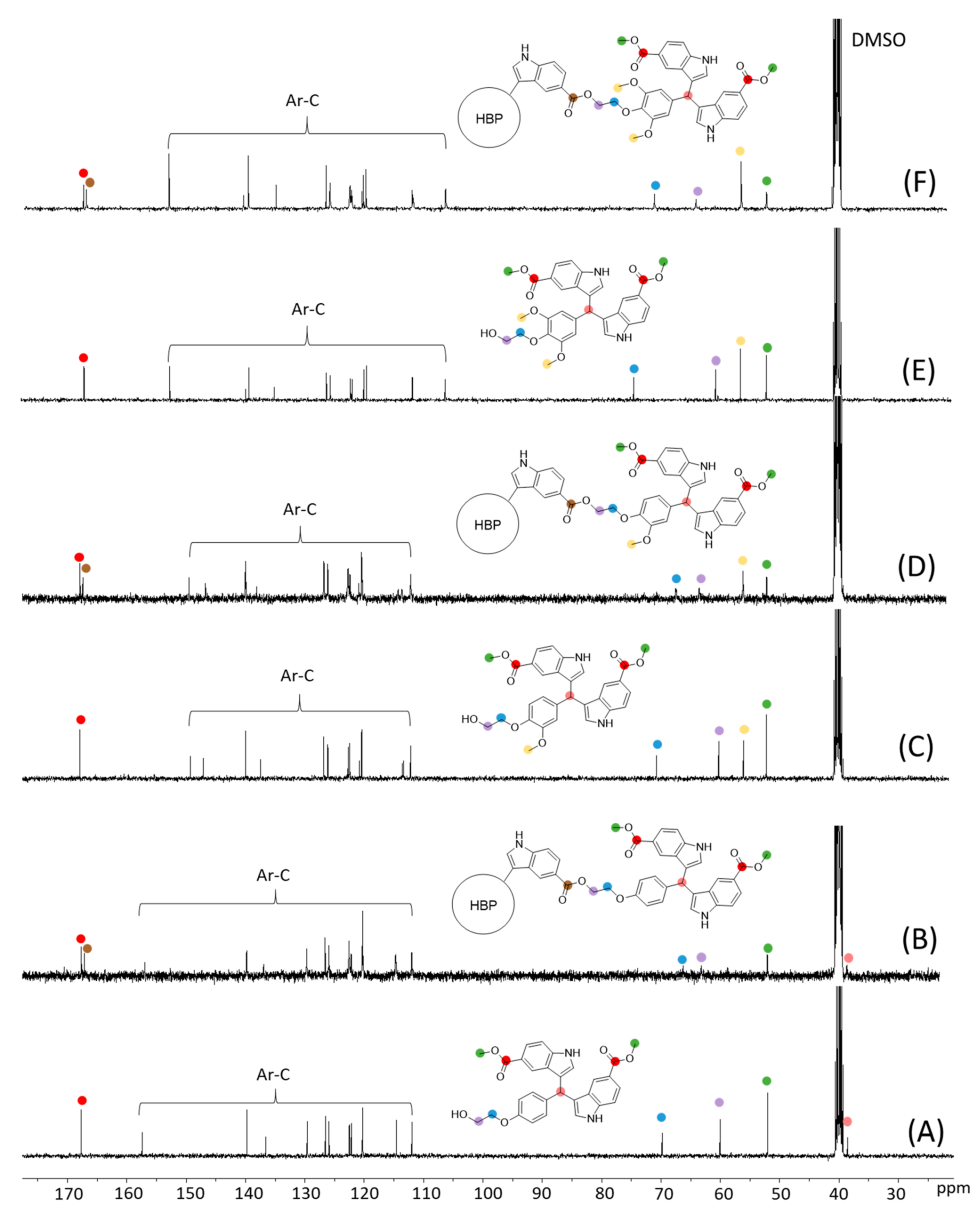
^13^C NMR spectra of (A) **5a**, (B) **P5a**, (C) **5b**, (D) **P5b**, (E) **5c**, and (F) **P5c** in DMSO-*d*_6_.

Next, ^13^C NMR spectroscopy provided further structural information about the synthesized monomers and polymers (Figure 2). The carbon signals for all the monomers were unambiguously assigned first (Figure 2A,C,E), including the ester carbonyl carbon (167.67, 167.67, and 167.68 ppm for **5a–c**, respectively), the aromatic carbons (∼153.15–106.48 ppm), the two ethylene “bridge” carbons (70.54, 69.81, and 74.52 ppm next to the aromatic ether unit and 60.07, 60.08, and 60.67 ppm next to the OH group for **5a–c**, respectively), the methyl ester carbons (∼52.05 ppm), the methoxy carbons (55.91 and 56.43 ppm for **5b–c**, respectively), and the signal for the central CH (38.61, 38.86, and 39.96 ppm for **5a–c**, respectively, confirmed by their HMQC spectra (Figures S4, S6, and S8, Supporting Information). After polymerizations, the signals for unreacted (end) carbonyl carbons (∼167.7 ppm), aromatic carbons, and the methyl carbons did not shift noticeably. Interestingly, the two ethylene “bridge” carbon signals showed the opposite trend of chemical shifts after the polymerizations. The one close to ester groups shifted downfield (by ∼3.22–6.15 ppm), but the other bridge carbon close to the phenoxy group shifted upfield (by ∼2.33–3.57 ppm). Additionally, a new signal at ∼167.2 ppm was observed in the ^13^C NMR spectra of the polymers, which corresponded to the carbonyl carbons of ester groups, indicating the formation of ester bonds in the polymers. The central CH carbon signal of **P5a** was observed at 38.62 ppm, but the same signal was not observed for **P5b–c**, due to overlapping with the DMSO signal at ∼40.61–39.36 ppm.

**Figure 3:**
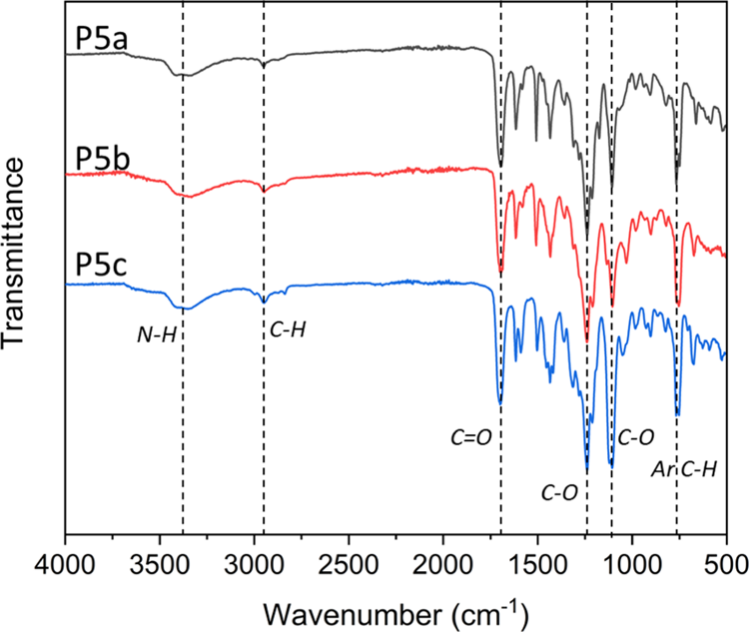
FTIR spectra of **P5a–c**.

In addition, the obtained HBPs were also characterized by FTIR spectroscopy (Figure 3). The characteristic absorption bands of **P5a–c** include indole N–H stretching (centered at ∼3390 cm^–1^), aliphatic C–H stretching (centered at ∼2952 cm^–1^), ester C=O stretching (∼1698 cm^–1^), C–O symmetric stretching (∼1239 cm^–1^), asymmetric C–O stretching (1106 cm^–1^), and aromatic C–H bending (∼761 cm^–1^) bands. Similar absorption bands were also observed in the FTIR spectra of the monomers **5a–c** (Figure S9, Supporting Information).

### 3.3 Thermal Properties

**Figure 4:**
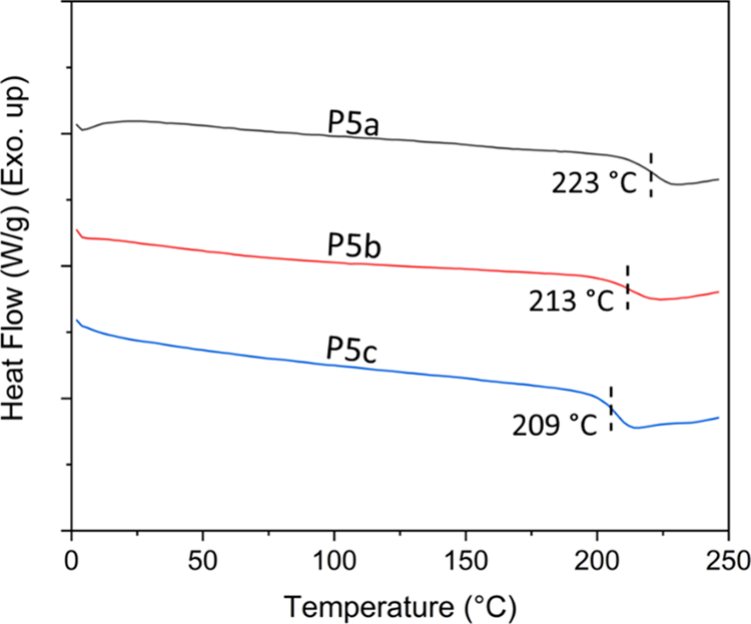
DSC second heating curves of **P5a–c**.

**Figure 5:**
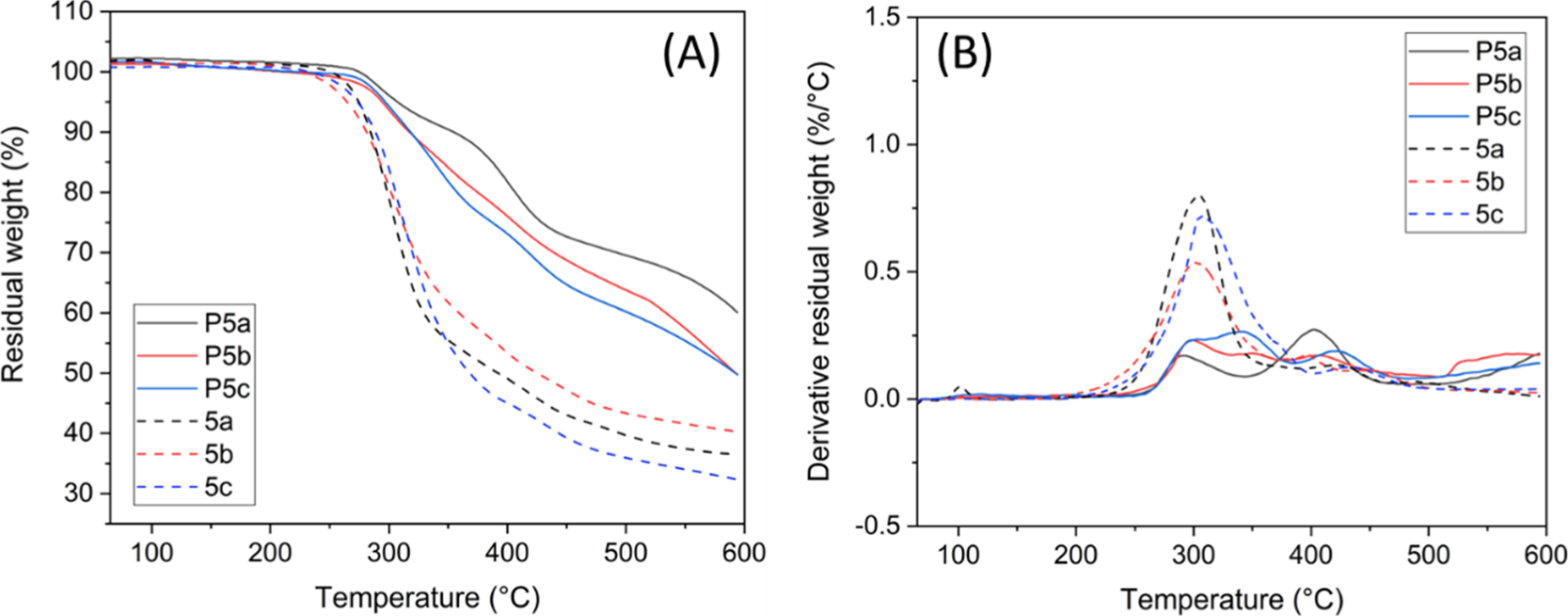
TGA residual weight (A) and first derivative (B) curves of monomers and polymers.

Thermal properties of **P5a–c** were characterized by DSC and TGA analyses. As shown in Figure 4, **P5a–c** showed high glass transition temperatures (*T*_g_ = 223, 213, and 209 °C, respectively), which was consistent with their rigid structures. The *T*_g_ values for **P5a–c** decreased upon the increasing number of methoxy groups in polymer structures, which could be related to the flexibility and plasticizing effect of the methoxy groups, as well as the slightly decreased molecular weight from **P5a** to **P5c**. No melting endotherm was observed, which revealed their fully amorphous nature. According to the TGA results (Figure 5), all the three HBPs showed relatively high initial thermal decomposition temperatures (*T*_10_ > 300 °C), which were higher than that of the corresponding monomers (*T*_10_ = 284, 283, and 286 °C for **5a–c**, respectively). Such enhanced thermal stability of polymers compared to their monomers was commonly observed for other HBPs.^16,37^ The derivative TGA curves showed multiple decomposition rate maxima. The first one at ∼290–325 °C could be attributed to the monomeric structures in the polymers, which was confirmed by the curves of the monomers (Figure 5B). The other decomposition rate maxima were observed at higher temperatures, which could be attributed to the degradation of the polyester backbones. The high residual char yields (CYs) of **P5a–c** (60, 50 and 50%, respectively) could be ascribed to the presence of aromatic structures, which indicated a potential inherent flame retardance.^67,68^

### 3.4 Antibacterial Effects

**Figure 6:**
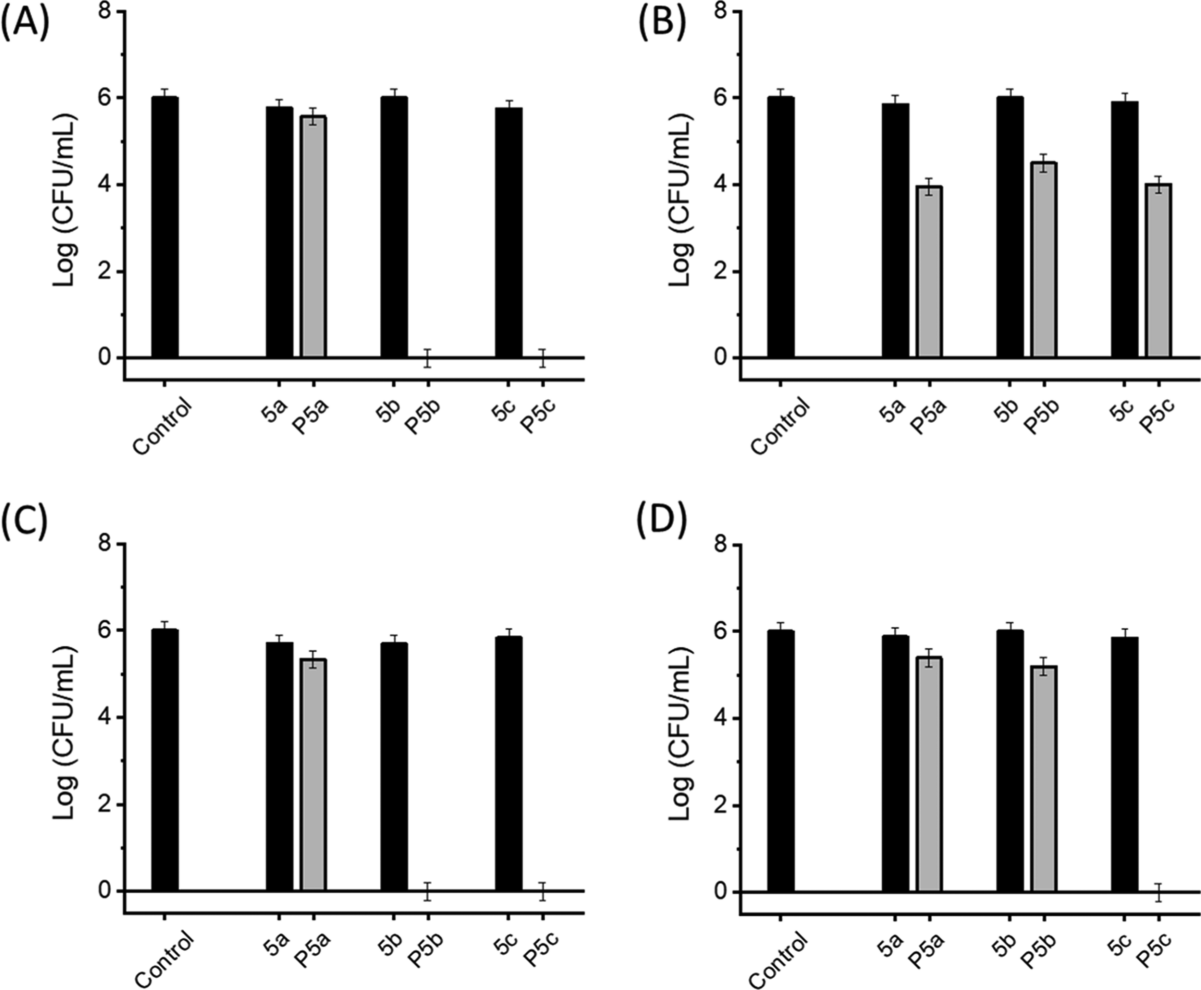
Colonies of Gram-negative bacteria (A) *E. coli* and (B) *P. aeruginosa* and Gram-positive bacteria (C) *S. aureus* and (D) *E. faecalis* on the surfaces coated with monomers (**5a–c**) or polymers (**P5a–c**). The control is an uncoated silicon wafer.

To evaluate the antibacterial activity, monomers **5a–c** and HBPs **P5a–c** were spin-coated on silicon substrates and tested against two Gram-negative (*E. coli* and *P. aeruginosa*) and two Gram-positive bacteria (*S. aureus* and *E. faecalis*) according to a conventional contact protocol.^60−62^ After confrontation with four pathogens for 24 h, the surviving bacteria were plated on a TSA Petri dish with 100-fold serial dilutions and incubated at 37 °C for another 24 h, as shown in Figure S10, Supporting Information. The antibacterial effects of the polymers and monomers were compared by calculating the number of viable bacterial colonies. As presented in Figure 6, polymers generally showed higher bactericidal activity compared to monomers (**5a–c**), which could be ascribed to their densely grafted functional groups (i.e., indole units) that can enhance their nonionic interactions with bacterial membranes. Such an enhancement of the antibacterial effect for HBPs was consistent with other reported HBPs.^8,16,37^ Specifically, **P5c** coating showed a significant antibacterial effect (∼6-log reduction in colony counts) against three of the selected bacteria (*E. coli*, *S. aureus,* and *E. faecalis*) and moderate antibacterial effect (∼2-log reduction in colony counts) against *P. aeruginosa*. **P5b** coating also showed a significant antibacterial effect of (∼6-log reduction in colony counts) against *E. coli* and *S. aureus* but a relatively low effect (∼1-log reduction in colony counts) against *P. aeruginosa* and *E. faecalis*. **P5a** coating exhibited only a moderate antibacterial effect (∼2-log reduction in colony counts) against *P. aeruginosa* but a rather insignificant effect (less than 1-log reduction in colony counts) against the other three bacteria. Based on such an observation, the increased number of methoxy substituents (**P5c** > **P5b** > **P5a**) on these HBP structures showed a general enhancement on the antibacterial effect. This was consistent with the observation with other cationic AMPs, for which the mild hydrophobic methoxy ether units could facilitate their interactions with bacterial membranes.^69^ However, there is still a general knowledge gap regarding the antimicrobial mechanism for nonionic AMPs, so the exact effect of methoxy ether units on nonionic AMPs remained to be unravelled.

**Figure 7:**
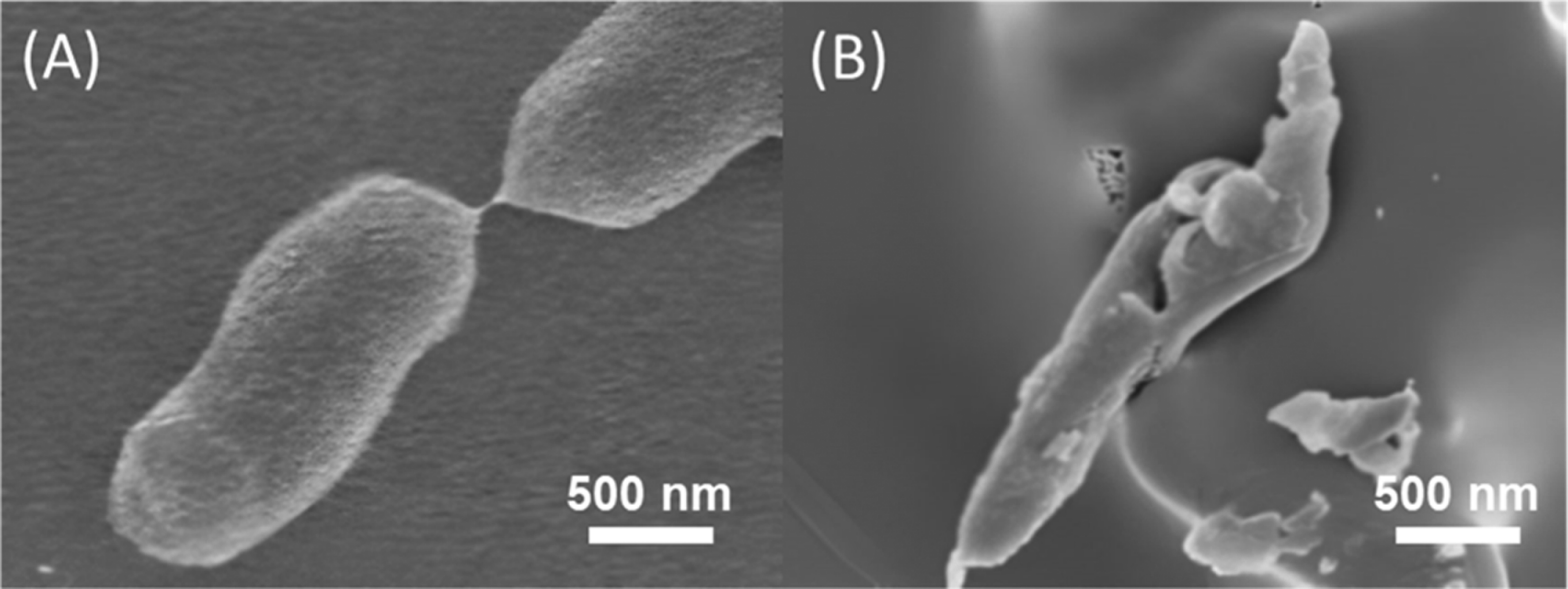
SEM images of *E. coli* before (A) and after (B) contacting a **P5c**-coated surface.

Next, the *E. coli* after contacting the **P5c**-coated surface was subjected to SEM imaging. As shown in Figure 7B, the cells of *E. coli* were clearly damaged after contacting **P5c** coating, which indicated the ability of **P5c** to disrupt bacterial membranes. This suggested a bactericidal mechanism, which was consistent with that of the other widely studied cationic AMPs.^70,71^

Furthermore, the coating thickness before and after the antimicrobial experiments against *E. coli* was investigated by ellipsometry. As shown in Table S2 (Supporting Information), the three polymer coatings were initially significantly thicker than the monomer coatings, which could be attributed to the superior film-forming ability of polymers compared to small molecules. It was also noted that **P5a** coating was thicker than the other two polymer coatings, which could be ascribed to its less hydrophobic structure (without hydrophobic methoxy units) and thus high affinity to the hydrophilic surface of silicon wafer. After the antimicrobial experiments against *E. coli*, the film thickness of polymers was only slightly reduced by approximately 1–6 nm, which indicated desirable film stability under the measurement conditions. SEM images of the polymer-coated surfaces before and after the antimicrobial experiments against *E. coli* indicated no observable difference (Figure S11, Supporting Information), which further confirmed the stability of the polymer coatings. Interestingly, no bacteria (no matter live or dead) was found in the SEM images of any polymer-coated surfaces after the antimicrobial experiments followed by simple water washing, which suggested the anti-fouling effect of these coatings.

In addition, the **P5c**-coated substrate was washed and dried overnight after the antimicrobial experiment (first cycle), and the obtained coating was again subjected to antimicrobial investigations against *E. coli* (second cycle). As shown in Figure S12, Supporting Information, no significant difference between the results of the two cycles was observed, which indicated the desirable stability and durability of **P5c** coating. On the contrary, no monomer formed stable films on the substrate, of which the film thickness or antimicrobial effect became immeasurable after the first cycle antimicrobial experiments.

It should be noted that the impact of different molecular weights on the observed antimicrobial effects was not investigated in this work. The molecular weights of the three obtained polymers were all in a similar range (they were polydisperse and not identical); so for these polymers, we consider the impact of different molecular weights insignificant. In addition, it has also been reported that the impact of the molecular weight of highly branched polymers on their antimicrobial effect was less significant due to their more compact globular structures compared to linear polymers.^36^ In the future, synthetic investigations on the methodologies to control the molecular weight and distributions are expected to facilitate a deeper understanding on the molecular weight effects.

### 3.5 Evaluation of Leaching

**Figure 8:**
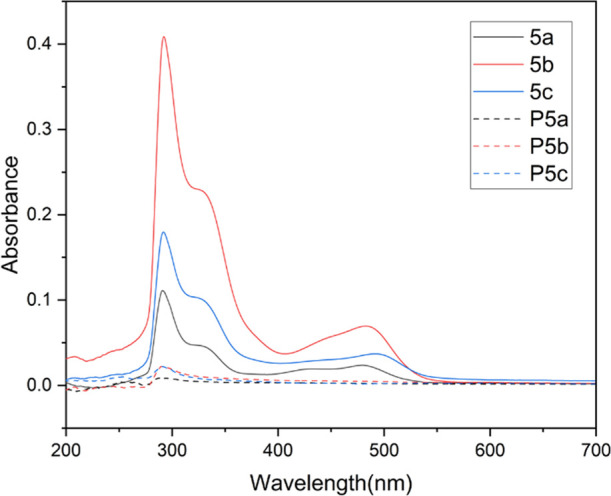
UV–vis absorbance spectra of the aqueous phase after the silicon wafers coated with monomers (**5a–c**) or HBPs (**P5a–c**) were immersed in deionized water for 5 days. The UV–vis spectra of the solutions of monomers and polymers in DMSO/H_2_O (1:9 v/v) were measured as references (Figure S13).

The release of antimicrobial agents from coatings may be hazardous to human health and environment, so antibacterial coatings without significant leaching will be desired.^50,60,72^ First, the prepared coatings were immersed in water for 5 days, and the aqueous phase was subjected to UV–vis measurements (Figure 8). As a result, negligible UV–vis absorbance was observed for the aqueous phase in which all three polymer coatings were immersed for 5 days. On the contrary, more significant UV–vis absorbance was observed for the aqueous phase with monomer coatings. These observations indicated a low leaching potential of polymers into the aqueous environment in 5 days.

**Figure 9:**
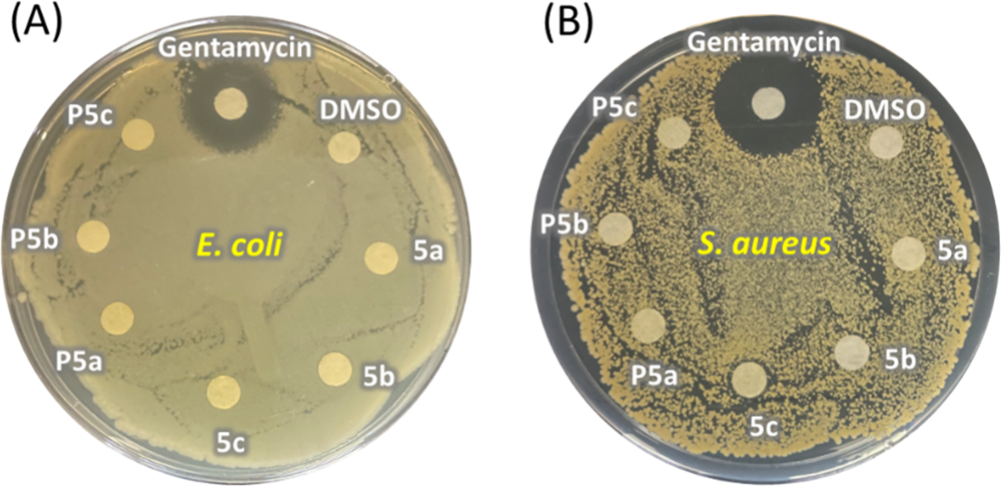
Photos of disk diffusion measurements of monomers (**5a–c**) and HBPs (**P5a–c**) in (A) *E. coli-* and (B) *S. aureus*-cultured lawns. Gentamycin and DMSO were used as controls. No zone of inhibition was observed around the disks containing monomers or polymers.

The general nonleaching nature of the HBP into aqueous environment was also demonstrated by disk diffusion measurements against *S. aureus* and *E. coli*. As a result (Figure 9), no zone of inhibition was observed around the disks containing monomers **5a–c** or polymers **P5a–c**, which indicated that these agents (when adsorbed on filter papers, not as coatings) did not leach out into the aqueous environment. Such a nonleaching nature could be attributed to the hydrophobicity of monomers and polymers, as well as the large size and low diffusion rate of polymers. In contrast, a significant zone of inhibition was clearly observed around the antibiotic gentamycin.

### 3.6 Hemotoxicity

**Figure 10:**
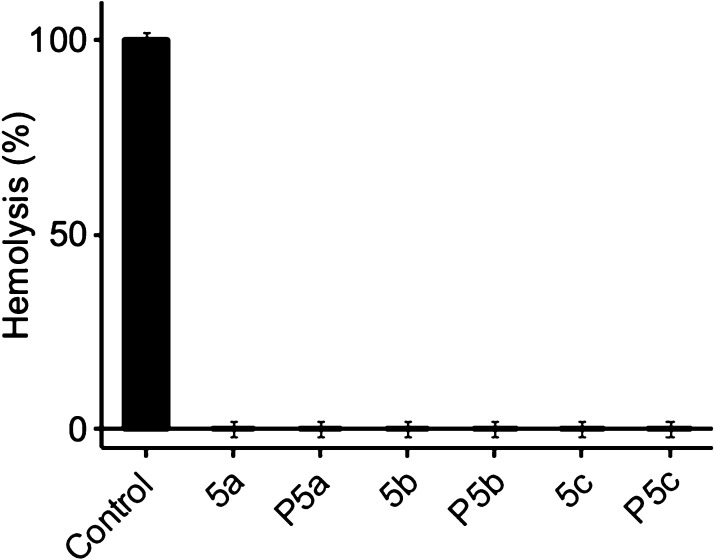
Hemolytic activity of monomers (**5a–c**) and HBPs (**P5a–c**). RBCs lysed with distilled water were used as the positive control.

Hemocompatibility of monomers **5a–c** and HBPs **P5a–c** was evaluated. A hemolysis test is a method to evaluate *in vitro* toxicity of materials on RBCs, which is important for any biomedically applied materials.^9,73,74^ As shown in Figure 10, the hemolysis rate of all the monomers and polymers were negligible (less than 0.1%) after 2 h of cultivation, demonstrating the hemocompatibility of these monomers and the corresponding HBPs and suitableness for potential biomedical applications. A similar effect for cationic polymers to selectively kill bacteria cells without killing RBCs has been reported before, which could be attributed to the different structures of bacterial and mammalian membranes.^69^

### 3.7 Cytotoxicity

**Figure 11:**
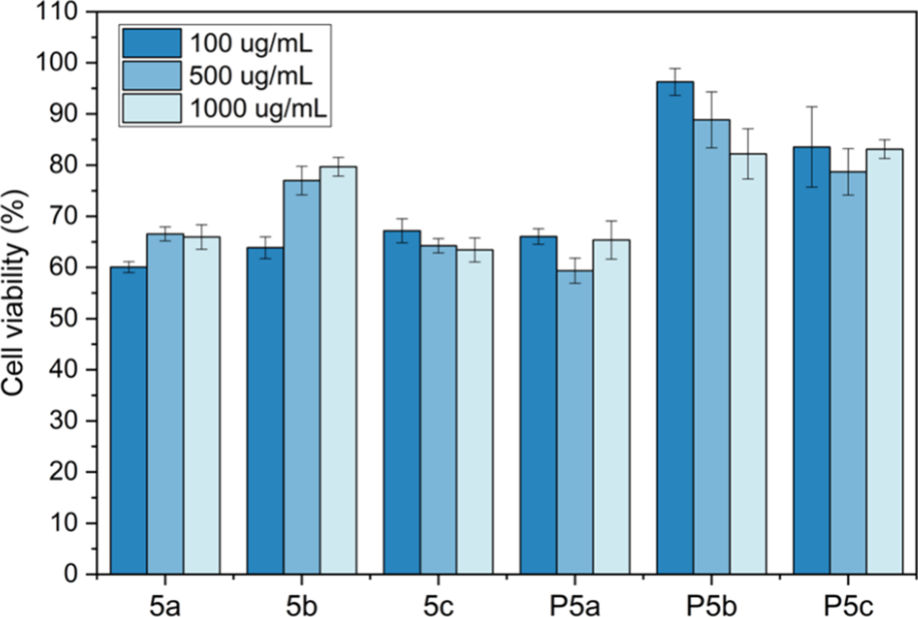
Cytotoxicity of monomers (**5a–c**) and polymers (**P5a–c**) against osteoblast-like human cells at three concentrations (100, 500, and 1000 μg/mL). The results were presented as the relative percent viability of the treated cells compared to that of the untreated control (100% of cell viability, not shown in the graph).

The biocompatibility of monomers **5a–c** and polymers **P5a–c** to MG-63 osteoblast-like human cells was further evaluated according to a standard MTT assay method. The results were presented as a relative percentage of the negative control (100% of cell viability). As illustrated in Figure 11, more than 30% of reduction of cell viability was observed for all the tested samples except for the two polymers with methoxy groups (**P5b** and **P5c**), which indicated that only **P5b** and **P5c** were noncytotoxic according to the ISO 10993-5 standard.^75^ This suggested that the methoxy ether groups improved the biocompatibility of the indole-based HBPs, which was consistent with other reported polymers.^69,76^

## 4 Conclusions

An indole carboxylate and three lignin-based monomeric aromatic aldehydes were used to synthesize a series of AB_2_ monomers with varied numbers of methoxy substituents. These monomers were polymerized to yield three nonionic HBP with a medium–low-molecular weight. The obtained nonionic polymers showed relatively high glass transition temperatures (*T*_g_ > 200 °C), good thermal stability (*T*_10_ > 300 °C), and desirable solubility in organic solvents. Furthermore, these polymers were conveniently coated on the silicon substrate by a solution spin-coating process, and the resulting polymer coatings showed significant bactericidal effects against two Gram-positive and two Gram-negative bacteria, as well as negligible leaching into an aqueous environment. Interestingly, we discovered that the antibacterial effect was enhanced with the increased number of methoxy ether units. Moreover, hemolysis and MTT assays revealed that the resulting polymers with methoxy groups showed desirable biocompatibility with RBCs and osteoblast-like human cells, indicating their potential in biomedical applications.

## Supporting Information Available

Solubility data of **P5a–c**; analytical data of grafting agents **3a–c** (^1^H NMR and ^13^C NMR spectra); analytical data of monomers **5a–c** (COSY spectra, HMQC spectra, and FTIR spectra), antibacterial images of **P5a–c**; antibacterial data of two cycles of tests of **P5c** against *E. coli*, SEM images, and thickness of **P5a–c** coatings before and after antibacterial experiments against *E. coli* (PDF)
