# Supplementary material for: Hyperbranched Polyesters Based on Indole- and Lignin-Derived Monomeric Aromatic Aldehydes as Effective Nonionic Antimicrobial Coatings with Excellent Biocompatibility
Source: Biomacromolecules. 2021 Dec 21;23(1):150–62. doi: 10.1021/acs.biomac.1c01186 (PMC8753607; doi:10.1021/acs.biomac.1c01186)
Supplement: Supplementary file 1 — bm1c01186_si_001.pdf [file bm1c01186_si_001.pdf]

Supporting Information

Hyperbranched polyesters based on indole and lignin-derived monomeric aromatic aldehydes as effective nonionic antimicrobial coatings with excellent biocompatibility

Xiaoya Li,[a] Xiao Wang,[b] Sathiyaraj Subramaniyan,[a] Yang Liu,[c] Jingyi Rao,[b] Baozhong Zhang*[a]*

[a] Centre for Analysis and Synthesis, Department of Chemistry, Lund University, P. O. Box 124, SE-22100 Lund, Sweden

[b] Hubei Key Laboratory of Material Chemistry and Service Failure, Hubei Engineering Research Centre for Biomaterials and Medical Protective Materials, School of Chemistry and Chemical Engineering, Huazhong University of Science and Technology, Wuhan, Hubei 430074, People's Republic of China

[c] Faculty of Medicine, Department of Clinical Sciences, Orthopedics, Lund University, SE-22184 Lund, Sweden

Table S1. Solubility of the obtained HBPs. + means soluble at room temperature, (⊕) means partially soluble at 60 °C, − means insoluble at 60 °C.

	Solvent	**P5a**	**P5b**	**P5c**
Aprotic solvents	Chloroform	–	–	–
	DCM	–	(⊕)	(⊕)
	1,4-Dioxane	(⊕)	(⊕)	(⊕)
	THF	+	+	+
	Acetone	(⊕)	(⊕)	(⊕)
	Acetonitrile	(⊕)	(⊕)	(⊕)
	DMF	+	+	+
	DMSO	+	+	+
	DMA	+	+	+
Protic solvents	Ethanol	(⊕)	(⊕)	(⊕)
	Methanol	(⊕)	(⊕)	(⊕)
	Water	–	–	–

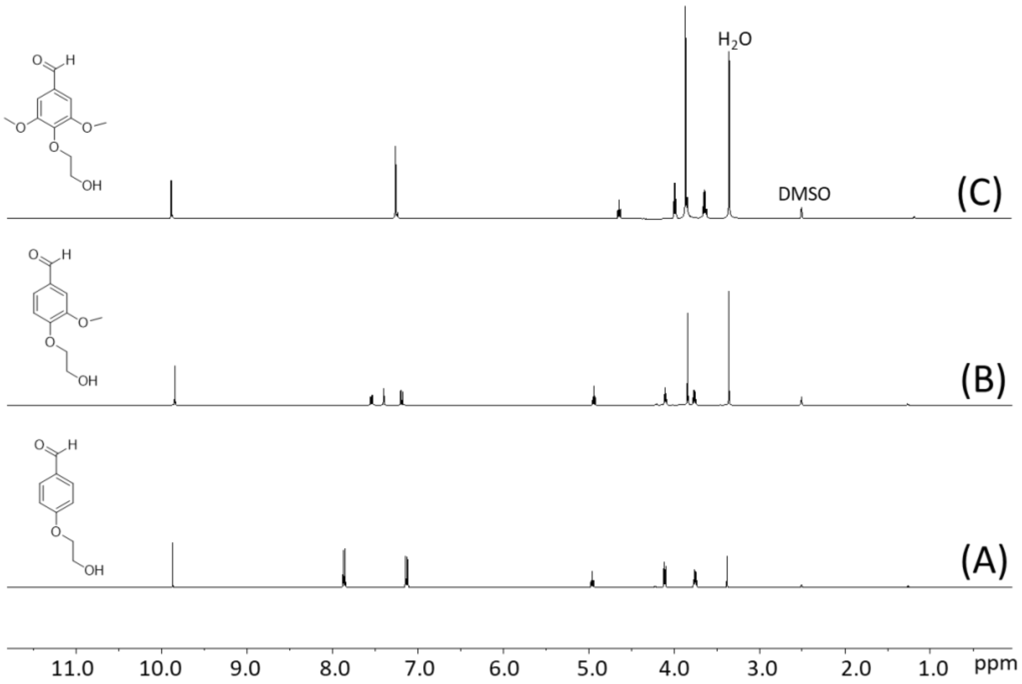

Figure S1. ^{1}H NMR spectra of (A) **2a**, (B) **2b**, and (C) **2c**.

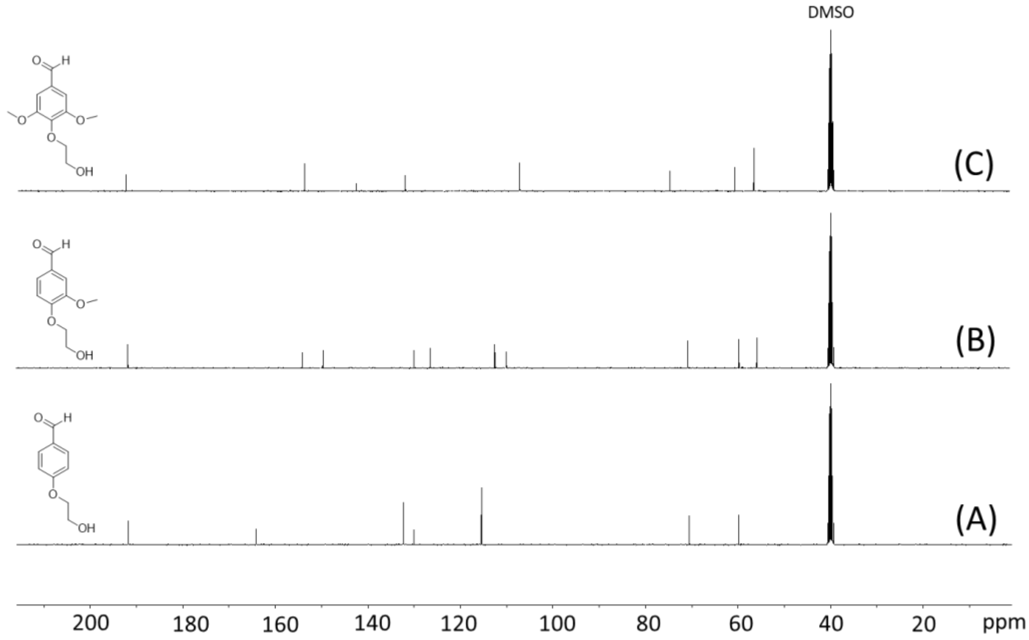

Figure S2. ^{13}C NMR spectra of (A) **3a**, (B) **3b**, and (C) **3c**.

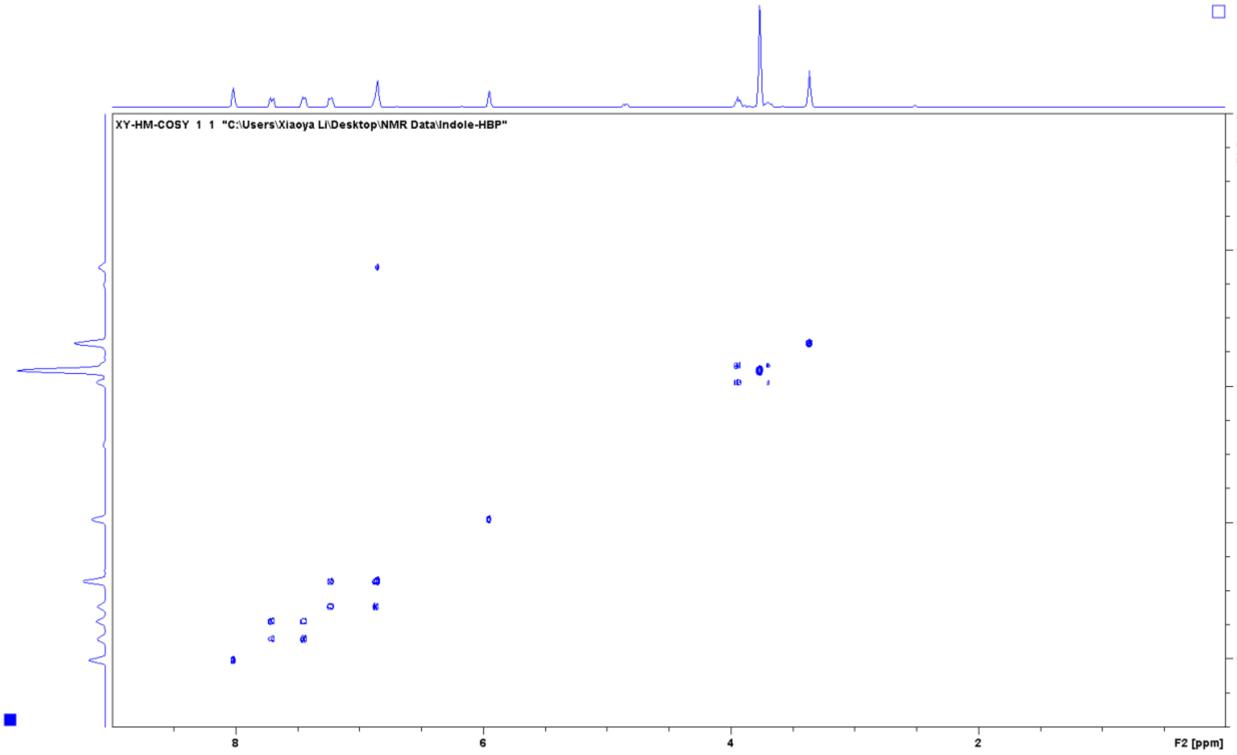

Figure S3. COSY spectrum of **5a**.

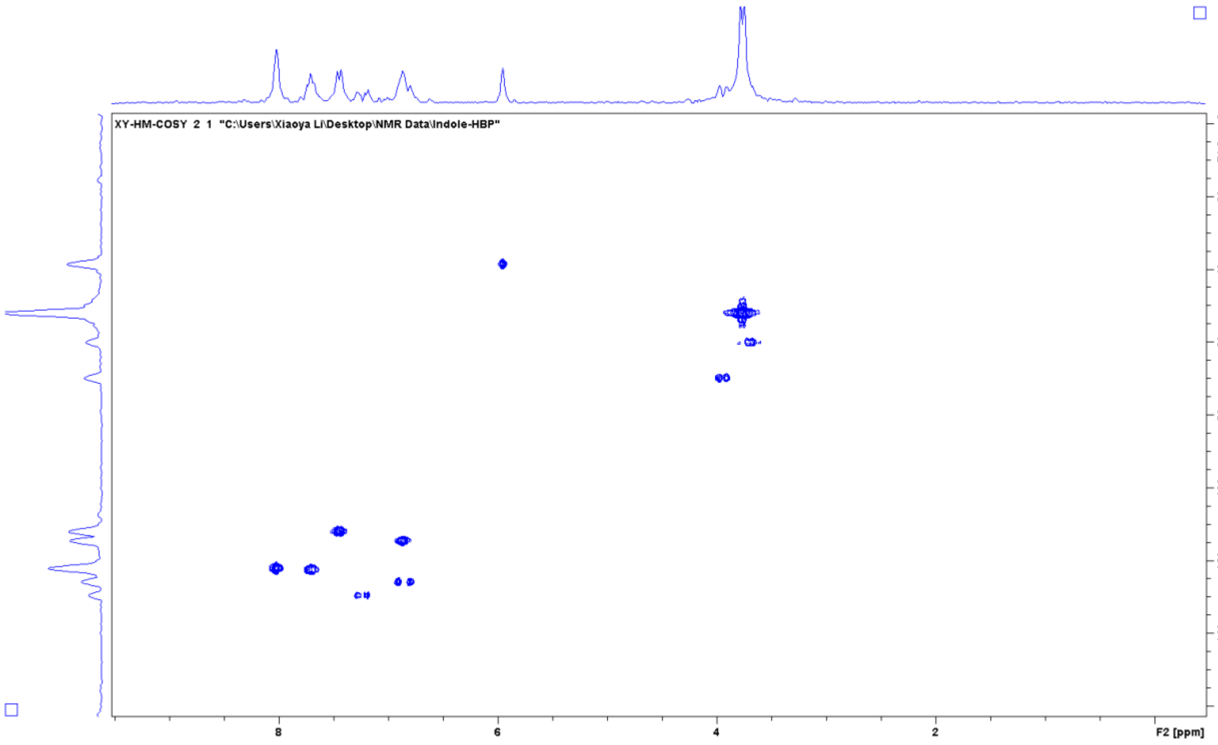

Figure S4. HMQC spectrum of **5a**.

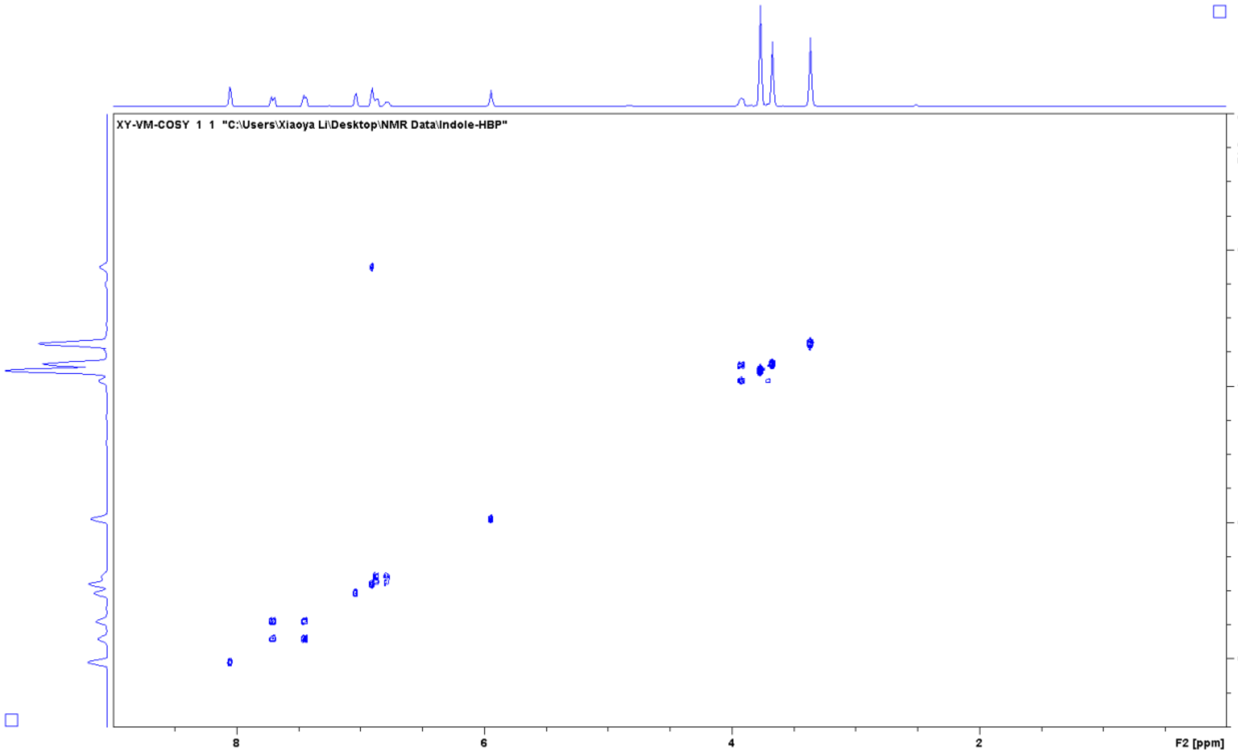

Figure S5. COSY spectrum of **5b**.

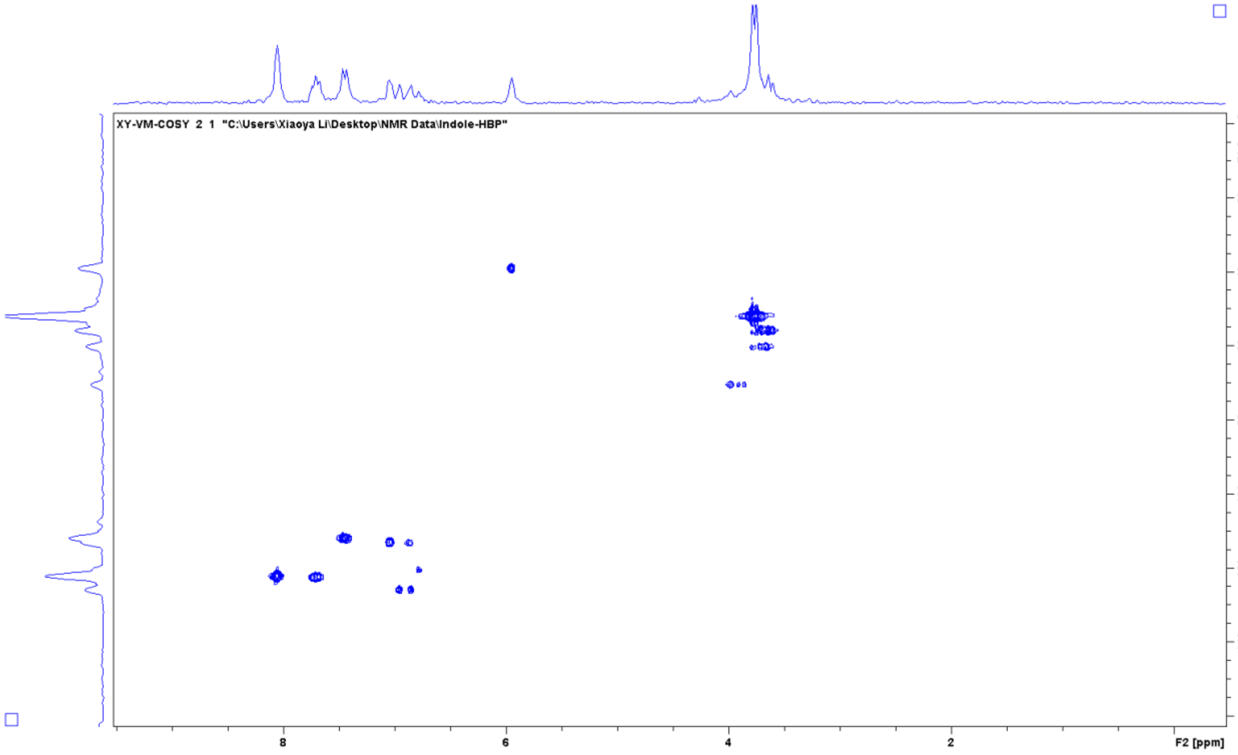

Figure S6. HMQC spectrum of **5b**.

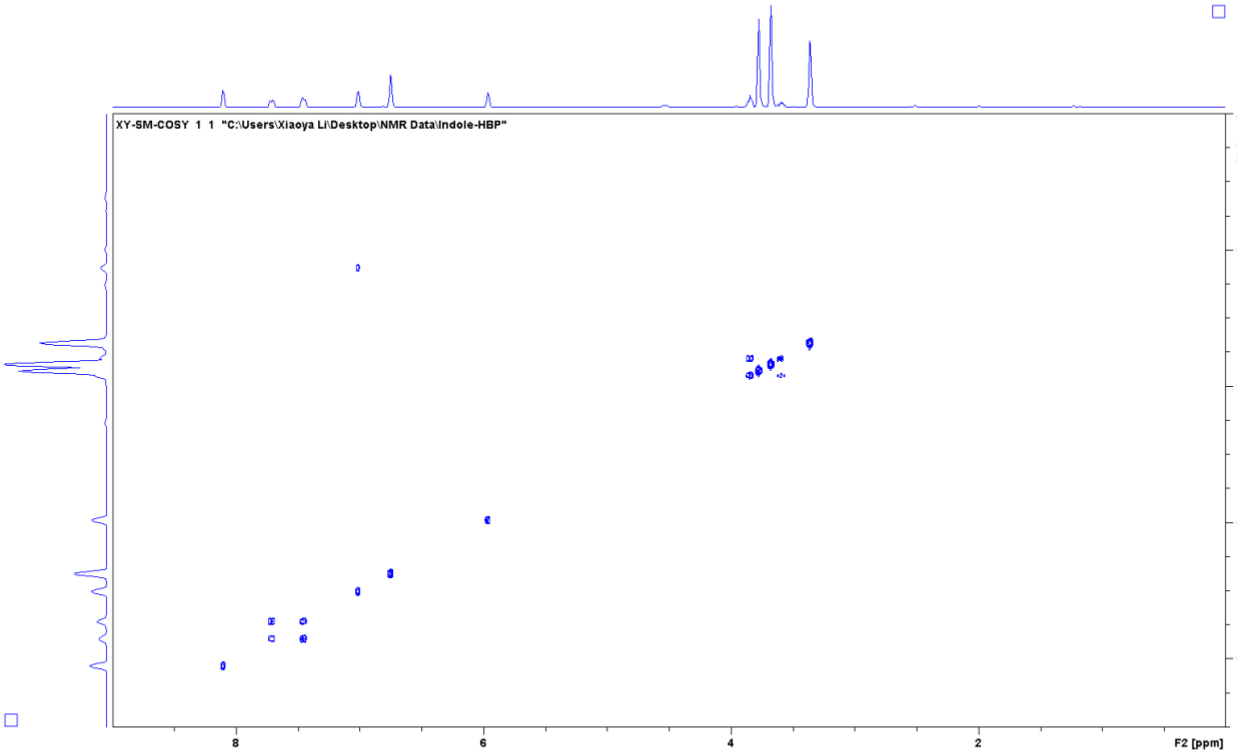

Figure S7. COSY spectrum of **5c**.

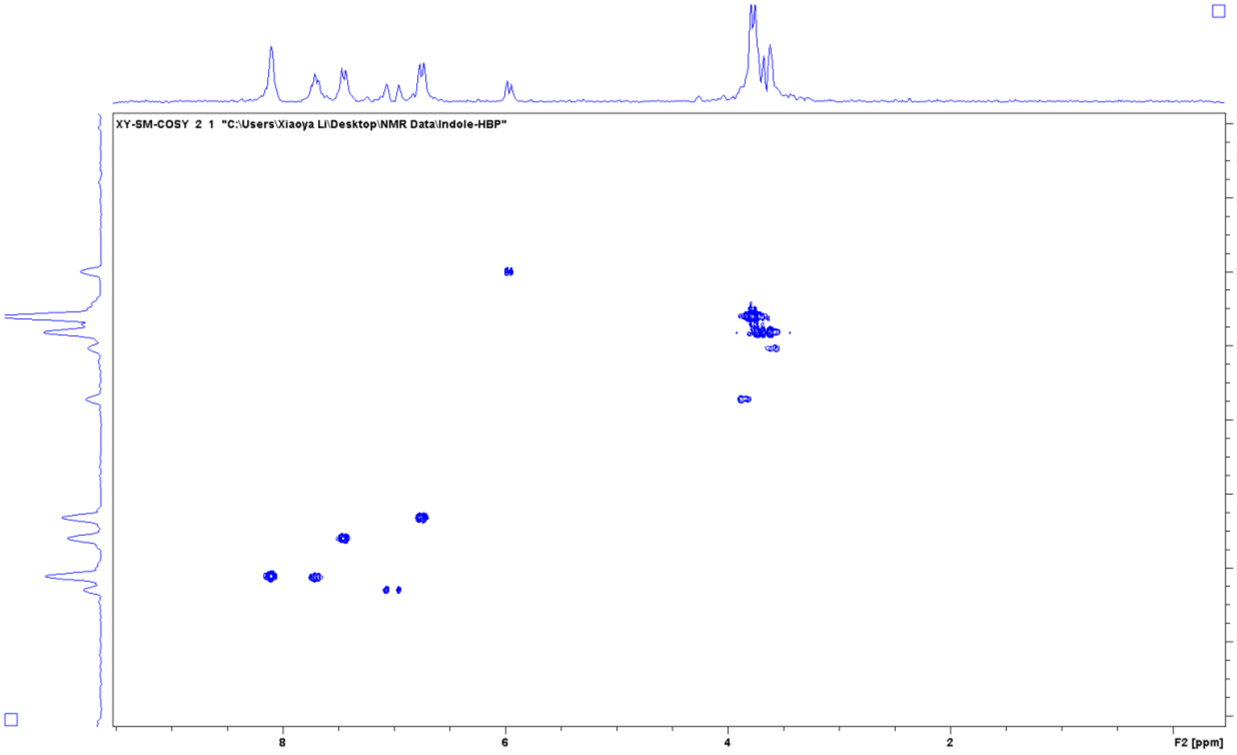

Figure S8. HMQC spectrum of **5c**.

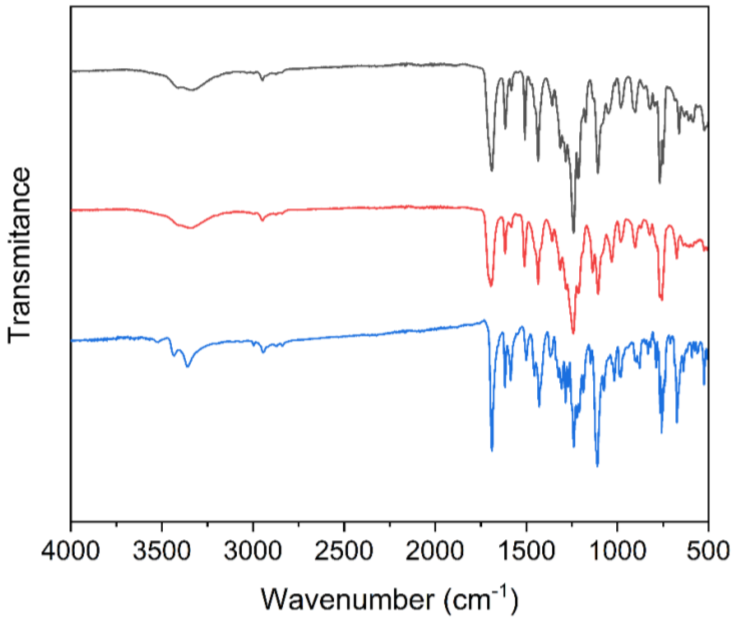

Figure S9. FTIR spectra of **5a-c**.

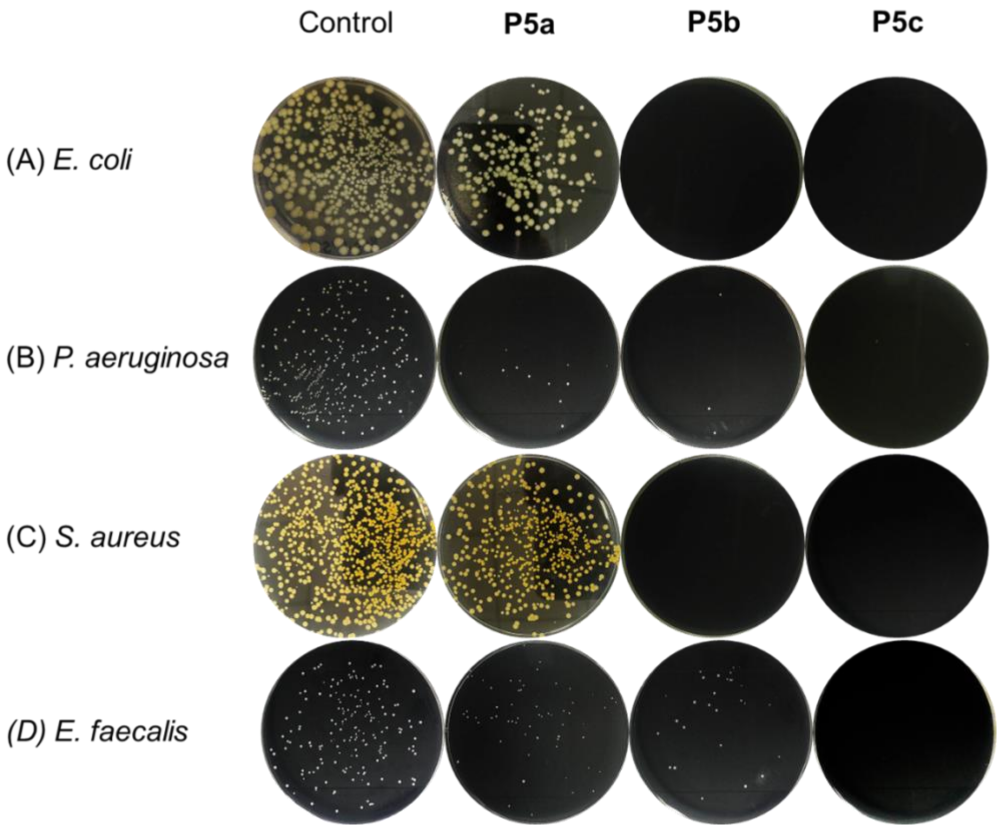

Figure S10. Petri-plates images of **P5a-c** against (A) *E. coli*, (B) *P. aeruginosa*, (C) *S. aureus* and (D) *E. faecalis*.

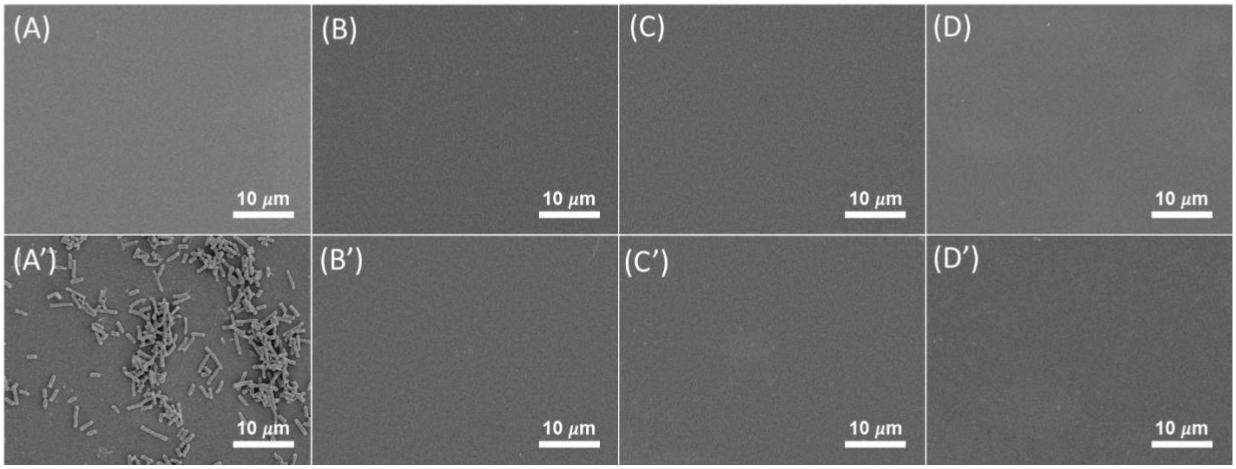

Figure S11. SEM images of (A) uncoated surface, (B) **P5a**, (C) **P5b** and (D) **P5c** coated surfaces before antibacterial experiments, and (A') uncoated surface, (B') **P5a**, (C') **P5b** and (D') **P5c** coated surfaces after antibacterial experiments against *E. coli*.

Table S2. The thickness of **P5a-c** coatings before and after antibacterial experiments against *E. coli* measured by ellipsometry.

Sample	Silicon wafer	5a	5b	5c	P5a	P5b	P5c
Thickness before (nm)	5.2	8.2	9.3	9.9	128.6	52.7	60.6
Thickness after (nm)	5.8	.	.	.	127.0	46.6	57.3

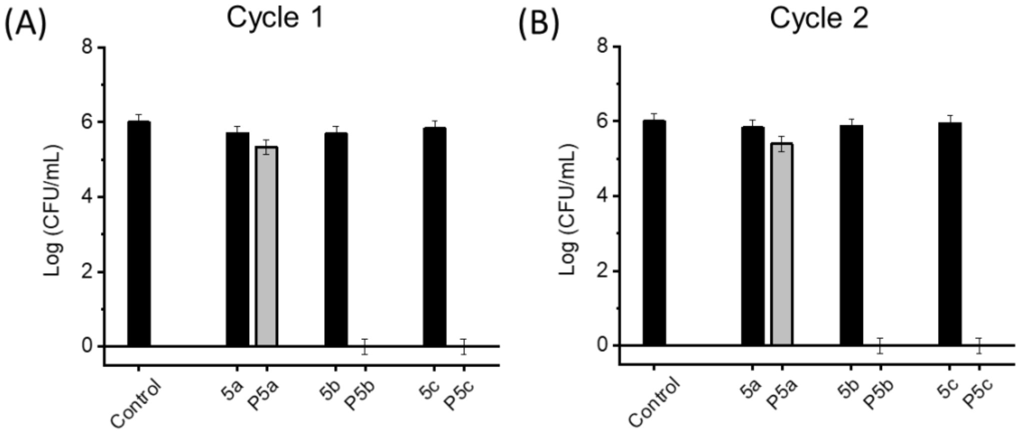

Figure S12. Durability of the antimicrobial activity of **P5c** against *E. coli*. Including the (A) first and (B) second cycle antimicrobial experiments.

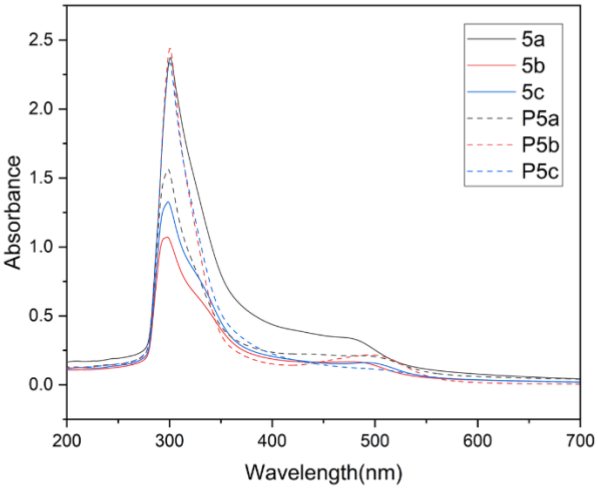

Figure S13. UV-vis absorbance spectra of the solutions of monomers (**5a-c**) and HBPs (**P5a-c**) in DMSO/H_2O (1 : 9, v/v) at 0.1 mg/mL.
